# Efferocytosis activates a DNMT3A-mediated oxidized DNA repair pathway to enable tissue resolution

**DOI:** 10.1172/JCI203265

**Published:** 2026-07-21

**Authors:** Kleopatra Avrampou, Santosh R. Sukka, David Ngai, Patrick B. Ampomah, Xiaobo Wang, George Kuriakose, Jacob Glass, Bernhard Dorweiler, Hanna Winter, Lars Maegdefessel, Hanrui Zhang, Aaron D. Viny, Ira Tabas

**Affiliations:** 1 Department of Medicine, Columbia University Irving Medical Center, New York, New York, USA.; 2 Center for Epigenetics Research, Memorial Sloan Kettering Cancer Center, New York, New York, USA.; 3 Department of Vascular and Endovascular Surgery, University of Cologne, Faculty of Medicine and University Hospital Cologne, Cologne, Germany.; 4 Institute of Molecular Vascular Medicine, TUM Klinikum, Technical University of Munich, Munich, Germany.; 5 Department of Physiology and Cellular Biophysics and; 6 Department of Pathology and Cell Biology, Columbia University Irving Medical Center, New York, New York, USA.

**Keywords:** Cell biology, Vascular biology, Atherosclerosis, Macrophages

## Abstract

Efferocytosis, the clearance of apoptotic cells by macrophages, promotes tissue resolution. Efficient resolution requires efferocytosis-induced macrophage proliferation (EIMP) to expand pro-resolving macrophages. Here, we show that efferocytosis activates base excision repair (BER) to remove 8-OHdG from DNA, enabling EIMP. Mechanistically, efferocytosis promotes poly(ADP-ribose) polymerase 1 (PARP1) chromatin binding and PARylation to facilitate DNA repair complex assembly and increases nuclear MutT homolog 1 (MTH1) (gene *NUDT1*), which hydrolyzes 8-OHdG. Both processes require DNA-methyltransferase-3A (DNMT3A), which is activated during efferocytosis. Using a model in which dexamethasone-induced thymocyte apoptosis triggers efferocytosis-mediated thymic repair, we showed that DNMT3A was required for increases in nuclear PARP1/MTH1, oxidized DNA suppression, EIMP in thymic macrophages, and thymic repair. We next studied a human-relevant model of atherosclerosis regression, whereby efferocytosis drove protective lesional fibrous cap thickening. We compared WT mice with a model of DNMT3A clonal hematopoiesis (CH), in which loss-of-function DNMT3A mutations promoted atherosclerotic disease. Atherosclerosis regression in WT mice led to decreased nuclear 8-OHdG and increased nuclear PARP1/MTH1 and EIMP in lesional macrophages and fibrous cap thickening, all of which were impaired in DNMT3A-CH regression. These findings reveal that efferocytosis initiates a BER pathway to allow macrophage proliferation for tissue resolution, with possible therapeutic relevance to atherosclerosis regression and DNMT3A-CH.

## Abstract 2

When macrophages clear dying cells, they repair DNA using a DNMT3A-dependent process to help macrophages multiply and heal tissue, which is required for atherosclerotic regression.

## Graphical abstract

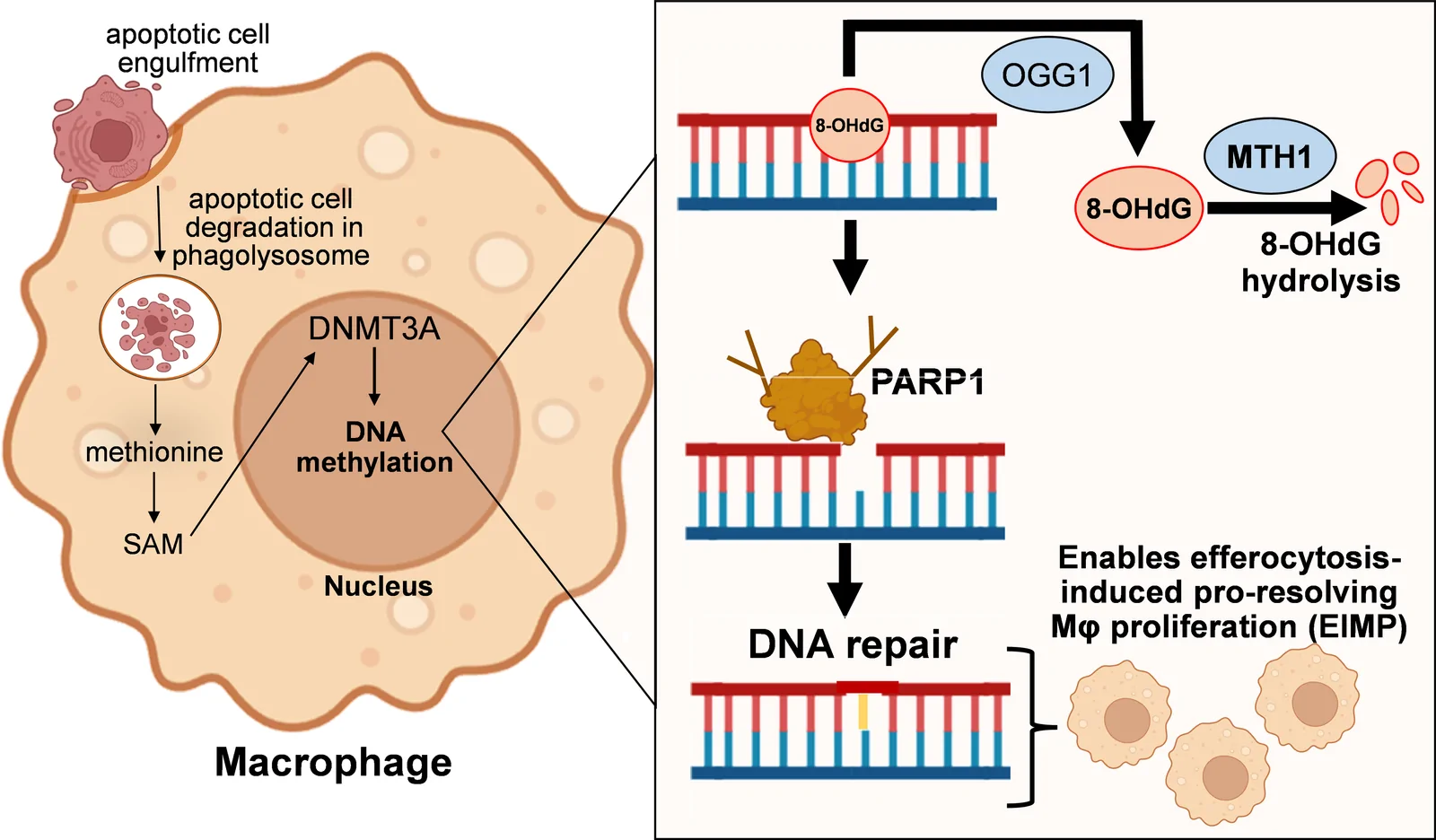

## Introduction

Efferocytosis, the process through which macrophages engulf and clear apoptotic cells (ACs), prevents secondary necrosis and promotes tissue resolution (1–3). Pathological impairment of efferocytosis-induced resolution drives many disease processes, including autoimmune, pulmonary, neurodegenerative, and atherosclerotic diseases (1–3). Efferocytosis triggers several pathways that reprogram macrophages to synthesize tissue repair factors, such as IL-10 and TGF-β1, and to acquire specialized features that enable the sequential uptake of multiple ACs, called continuing efferocytosis, which is critical for tissue repair in vivo (4–12). Several mechanisms linking efferocytosis to pro-resolving macrophage reprogramming have been identified, including efferocytosis receptor–mediated signaling pathways, alterations in macrophage energy metabolism, and pathways triggered by molecules derived from the phagolysosomal degradation of ACs (6–9, 12–15). For example, AC-derived methionine, after conversion to S-adenosylmethionine, activates DNA-methyltransferase-3A–mediated (DNMT3A-mediated) DNA methylation, which enhances a pro-resolving ERK1/2/TGF-β1 pathway in efferocytosing macrophages (8). As another example, AC-derived nucleotides trigger a Myc-mediated proliferation pathway, known as efferocytosis-induced macrophage proliferation (EIMP) (7, 15). EIMP expands the pool of pro-resolving macrophages to enhance continuous AC clearance and tissue repair in vivo (7, 15), and recent work has suggested a broad effect of EIMP in a variety of in vivo settings (16–22). In this context, identifying additional mechanisms linking efferocytosis to pro-resolving macrophage reprogramming and understanding how macrophages integrate distinct efferocytosis-induced reprogramming pathways to carry out effective resolution represent key goals in this critical area of biomedical research.

During studies aimed at identifying additional pro-resolving effects of the methionine/DNMT3A pathway, we made the unexpected observation that efferocytosis lowered nuclear 8-hydroxy-2′-deoxyguanosine (8-OHdG), a marker of oxidized DNA (oxDNA), and that this process required DNMT3A. Mechanistically, efferocytosis-induced DNA methylation by DNMT3A enhanced chromatin binding of poly(ADP-ribose) polymerase 1 (PARP1), facilitating base excision repair (BER) complex formation, while concurrently increasing nuclear MutT homolog 1 (MTH1) (gene *NUDT1*) to hydrolyze excised 8-OHdG. We demonstrate that this DNMT3A-mediated oxDNA suppression pathway occurs in efferocytosing macrophages in vivo and is required for EIMP, probably by enabling DNA synthesis, and facilitates tissue resolution. In particular, the pathway plays a key role in a human-relevant model of atherosclerosis regression induced by cholesterol lowering (23–27), whereby efferocytosis-driven resolution, including EIMP, promotes the formation of a protective fibrous cap over atherosclerotic plaques (6, 7, 9, 28). We now show the role of the DNMT3A/BER pathway in regression-induced cap thickening. For this purpose, we used a model of DNMT3A clonal hematopoiesis (CH), reflecting human clinical data showing that somatic DNMT3A loss-of-function (LoF) mutations are associated with an increased risk of atherosclerotic disease (29–40). We also found that lesional macrophages in unstable regions of human carotid atheroma displayed elevated levels of 8-OHdG and reduced nuclear PARP1 and MTH1 compared with stable regions.

Thus, we have identified what we believe to be a previously unrecognized mechanism linking efferocytosis to pro-resolving macrophage reprogramming involving a BER-induced oxDNA suppression pathway activated by DNMT3A-mediated DNA methylation. This pathway contributes to tissue resolution, including in the critical process of atherosclerosis regression, with possible relevance to DNMT3A-CH. The role of the pathway in EIMP suggests integration between 2 AC cargo–driven resolution pathways: one involving AC-derived methionine and DNMT3A and the other involving AC-derived nucleotides, together supporting the expansion of pro-resolving macrophages.

## Results

### Efferocytosis induces the suppression of nuclear oxDNA in a DNMT3A-dependent manner.

While studying responses of macrophages to the uptake of Jurkat cells rendered apoptotic by UV irradiation (ACs), we observed a previously unknown phenomenon in which efferocytosis suppressed basal levels of nuclear oxDNA. Specifically, we incubated mouse bone marrow–derived macrophages (BMDMs) in the absence or presence of ACs for 45 minutes, which stimulated efferocytosis, followed by AC removal and a 1-hour chase period. The macrophages were then immunostained for the oxDNA marker 8-OHdG and various nuclear markers. The data show that 8-OHdG MFI in nuclei, identified by immunostaining for histone 2A (H2A) or lamin B1, was suppressed by efferocytosis in macrophages (Figure 1, A and B). To understand how efferocytosis causes the suppression of nuclear oxDNA, we explored pathways known to be activated by AC uptake in macrophages. One such pathway involves AC-derived methionine, which increases the methyl donor S-adenosylmethionine (SAM) and SAM-dependent DNMT3A-mediated DNA methylation (8). We found that efferocytosis-induced suppression of nuclear oxDNA was not seen in BMDMs lacking DNMT3A (DNMT3A-KO) (Figure 1, A and B), which could not be explained by impaired primary efferocytosis in DNMT3A-KO versus control macrophages (Supplemental Figure 1A; supplemental material available online with this article; https://doi.org/10.1172/JCI203265DS1). Efferocytosis also decreased total cellular 8-OHdG in a DNMT3A-dependent manner after a 1-hour chase, and we also observed this suppression after a 3-hour chase but not after a 6-hour or 24-hour chase (Supplemental Figure 1B).

Moving beyond mouse BMDMs, we observed similar results when comparing efferocytosing human monocyte–derived macrophages (HMDMs) treated with siDNMT3A versus scrambled RNA (Figure 1C and Supplemental Figure 1C). Moreover, we also tested human apoptotic macrophages generated by staurosporine treatment, which reflects the major source of ACs in atherosclerosis (41) (see final section). The results were like those observed using apoptotic Jurkat cells: efferocytosis suppressed nuclear 8-OHdG in WT (control) but not DNMT3A-KO (Figure 1D) macrophages.

Our previous work showed that DNMT3A-mediated DNA methylation in efferocytosing macrophages was driven by a methionine/SAM pathway that utilized the methionine from the engulfed and degraded ACs (8). To test the relevance of this AC cargo pathway here, we pretreated macrophages with bafilomycin A1, a lysosomal vacuolar ATPase inhibitor that blocks the phagolysosomal degradation of ACs during efferocytosis (42–45). Inhibition of lysosomal degradation blocked the efferocytosis-induced suppression of nuclear oxDNA (Supplemental Figure 1D), indicating that AC degradation was required for this effect. Moreover, inhibition of SAM synthesis using the methionine adenosyltransferase 2A (MAT2A) inhibitor PF-9366 similarly blocked efferocytosis-induced suppression of nuclear oxDNA (Supplemental Figure 1E). Finally, as DNMT3A can affect cells by both DNA methylation-dependent and -independent mechanisms (42–45), we tested whether DNA methylation is required for efferocytosis-induced suppression of nuclear oxDNA. For this purpose, we used macrophages treated with the DNA methylation inhibitor 5-AzaC or transduced with the DNA demethylase Tet1. Both interventions blocked efferocytosis-induced suppression of oxDNA in WT macrophages (Figure 1, E and F). These combined data reveal a pathway triggered by efferocytosis, in which DNMT3A-mediated DNA methylation suppressed the basal level of 8-OHdG in macrophages. Below, we explore the mechanism of 8-OHdG suppression and, most important, its functional significance.

### Efferocytosis-induced suppression of oxDNA is dependent on PARP1 and MTH1.

We hypothesized that suppression of 8-OHdG by efferocytosis could result either from reduced ROS production or activation of DNA repair. Using an ROS-sensitive fluorogenic probe (CellROX), we found that ROS increased with efferocytosis, which is consistent with a previous study from our group (27), but DNMT3A deletion did not suppress efferocytosis-induced ROS (Supplemental Figure 2A). We therefore investigated whether efferocytosis, through DNMT3A, promotes DNA repair. To begin, we showed that the double-stranded break marker γH2AX was similar between DNMT3A-KO and control macrophages before and after efferocytosis (Supplemental Figure 2B), suggesting that repair of dsDNA breaks was not involved. We then explored a possible role for BER of oxDNA in efferocytosing macrophages. BER repair of oxDNA involves excision of 8-OHdG by 8-oxoguanine glycosylase (OGG1) followed by (a) repair of the excised DNA by a protein complex in which PARP1-mediated PARylation plays a key role by promoting recruitment of the DNA repair proteins; and (b) hydrolysis of the excised 8-OHdG by MTH1 to prevent 8-OHdG reincorporation into DNA during DNA replication (46, 47) (Supplemental Figure 2C).

In support of a role for BER, we showed that silencing OGG1 blocked efferocytosis-induced suppression of nuclear oxDNA (Figure 2A and Supplemental Figure 2D). Focusing next on PARP1, we found that its nuclear localization increased with efferocytosis in WT but not DNMT3A-KO macrophages (Figure 2B). Efferocytosis did not significantly alter total cellular *Parp1* mRNA or protein levels (Supplemental Figure 2, E and F). Most important, silencing PARP1 in WT macrophages blocked efferocytosis-induced suppression of oxDNA (Figure 2C and Supplemental Figure 2G). Similar results were observed using the PARP inhibitor *N*-(6-oxo-5,6-dihydro-phenanthridin-2-yl)-*N*,*N*-dimethylacetamides HCl (PJ34) (48), which inhibited PARP1 enzymatic activity and PAR chain formation, in both mouse BMDMs and HMDMs (Figure 2, D and E).

A key step in PARP1-mediated DNA repair is binding of PARP1 to chromatin and protein PARylation, which induces PARP1 enzymatic activation and PARylation of PARP1 and associated proteins (49). Accordingly, we found that both PARP1 chromatin binding and PARylation activity were increased by efferocytosis in WT macrophages, but much less so in DNMT3A-KO macrophages (Figure 2F). To link to DNA methylation, we first showed that efferocytosis-induced nuclear PARP1 was accompanied by an increase in 5-methylcytosine, associated with an increase in nuclear PARP1 (Supplemental Figure 2H). Further, 5-AzaC treatment of macrophages blocked efferocytosis-induced PARP1 chromatin association as assessed by immunoblot (Figure 2G), and transduction of macrophages with Tet1 demethylase blocked efferocytosis-induced nuclear PARP1 as assessed by immunofluorescence microscopy (Figure 2H). Thus, efferocytosis-induced nuclear PARP1, like suppression of 8-OHdG, was mediated by the DNA methyltransferase activity of DNMT3A.

We next turned our attention to MTH1. As with PARP1, efferocytosis increased nuclear MTH1 in WT but not in DNMT3A-KO macrophages, as measured by immunofluorescence microscopy or immunoblotting of the nuclear soluble fraction in mouse and human macrophages (Figure 3, A–C). Efferocytosis did not significantly alter total cellular *Nudt1* mRNA or MTH1 protein levels (Supplemental Figure 3, A and B). Moreover, silencing MTH1 (siNudt1) or treatment with the MTH1 inhibitor TH287 (50) prevented the suppression of 8-OHdG in WT macrophages (Figure 3, D and E, and Supplemental Figure 3C). These effects, and those above related to siParp1 and PJ34, could not be explained by inhibition of primary AC uptake (Supplemental Figure 3, D and E). As with PARP1, efferocytosis-induced nuclear MTH1 in WT macrophages was inhibited by 5-AzaC and Tet1 transduction, supporting a role for DNA methylation (Figure 3, F and G). Most important, retroviral transduction of DNMT3A-KO macrophages with MTH1 corrected the defect in efferocytosis-induced suppression of oxDNA in these cells (Figure 3H and Supplemental Figure 3F). Collectively, these data indicate a pathway in which efferocytosis promoted the lowering of oxDNA through a DNMT3A-dependent, PARP1/MTH1-mediated DNA repair pathway.

### The suppression of oxDNA is required for efferocytosis-induced macrophage proliferation.

Efferocytosis-induced changes in macrophages are often linked to the activation of tissue resolution pathways, and thus we wondered if efferocytosis-induced suppression of oxDNA was required for a specific resolution process. We considered a key tissue resolution process called EIMP because EIMP requires DNA synthesis (7). EIMP, which is triggered by AC-derived nucleotides and Myc upregulation, expands the pool of pro-resolving efferocytic macrophages during tissue injury and is essential for the proper clearance of ACs and tissue repair in vivo (7, 15–22). Given that impaired repair of oxDNA can block cell proliferation in other settings (51), we hypothesized that efferocytosis-induced suppression of oxDNA might be necessary for EIMP when macrophages are exposed to ACs during tissue injury.

To explore this hypothesis in vitro, we incubated WT and DNMT3A-KO macrophages with ACs to induce efferocytosis. As previously reported (7), the number of WT macrophages increased when the macrophages were observed 24 hours later, but this was not observed with the DNMT3A-KO macrophages (Figure 4A). As additional markers of EIMP, we showed previously that efferocytosis in macrophages increases both the expression of the proliferation marker Ki67 and the incorporation of 5-ethynyl-2′-deoxyuridine (EdU) into DNA (7). Here, both endpoints were lower in DNMT3A-KO versus WT macrophages after AC exposure (Figure 4B), whereas Ki67 and EdU levels did not differ between genotypes in the absence of ACs (Supplemental Figure 4, A and B). A marker of oxDNA-induced cell-cycle arrest is the elevation of the cell-cycle checkpoint response protein p21^WAF1/Cip1^ (CDKN1A), which is an inhibitor of cyclin-dependent kinase (CDK) that arrests cells in the G_1_/G_2_ phase (52). We found that p21 expression following efferocytosis was higher in DNMT3A-KO macrophages than in WT macrophages, with no difference between macrophages incubated in the absence of ACs (Figure 4C and Supplemental Figure 4C), suggesting that failure of efferocytosis-induced suppression of oxDNA caused cell-cycle arrest. We also considered a role for p53 levels, as p21 is a known target of p53, but we found that the expression of p53 was not altered by efferocytosis, and pharmacological inhibition of p53 with pifithrin-α (PFTα) (53) did not affect p21 expression (Supplemental Figure 4, D and E). These data suggest that p21 was increased by a p53-independent mechanism (54) in efferocytosing DNMT3A-KO macrophages, and, given the role of p53 in dsDNA breaks (55), these data were consistent with the negative *γH2AX* data above.

We next tested the role of PARP1 and MTH1 in EIMP, i.e., as a link to the aforementioned DNMT3A-dependent oxDNA repair mechanism. We found that inhibition of PARP1 with PJ34 or MTH1 with TH287 blocked EIMP in WT mouse macrophages, as measured by cell numbers or Ki67 expression (Figure 4, D and E). PJ34 also lowered Ki67 expression in WT human macrophages (Figure 4F). Conversely, retroviral transduction of efferocytosing DNMT3A-KO macrophages with MTH1 increased Ki67 expression and the percentage of macrophages incorporating EdU into their DNA (Figure 4G). Thus, the DNMT3A/PARP1/MTH1-mediated oxDNA repair mechanism in efferocytosing macrophages was necessary for EIMP in vitro.

### Evidence of DNMT3A-mediated suppression of oxDNA in efferocytosing macrophages in vivo.

To determine whether DNMT3A-mediated suppression of oxDNA occurs when macrophages are exposed to ACs in vivo, we turned to a well-established model of efferocytosis-induced tissue resolution in which mice are injected with dexamethasone (DEX) (56). Within 4 hours of DEX injection, apoptotic thymocytes increase, which initiates efferocytosis by thymic macrophages (6, 56). This phase of efferocytosis reflects in vivo the process referred to as primary efferocytosis in vitro. Efferocytosis-induced repair processes then follow, and by 18 hours, tissue repair is well underway (6–9). However, mice with defective efferocytosis or impaired efferocytosis-induced resolution signaling show elevated thymic necrosis at the 18-hour time point (6–9). Efferocytosis observed at 18 hours is representative of continuing efferocytosis, which is the sequential clearance of multiple ACs by a single macrophage, and it is a critical process for tissue repair (4–9).

To begin, we transplanted irradiated WT mice with bone marrow from control *Vav1cre^+/–^* (control) or *Dnmt3a^fl/fl^Vav1cre^+/–^* mice to create hematopoietic DNMT3A–KO (H-DNMT3A–KO) mice. After 4 weeks of recovery, the mice were injected with PBS or DEX. Focusing first on the DEX-treated mice, we documented that DNMT3A expression in thymic macrophages was significantly diminished in the KO cohort (Figure 5A and Supplemental Figure 5A). Moreover, we verified the presence of efferocytosing macrophages in the thymus, measured as the ratio of macrophage-associated to free TUNEL^+^ cells (Figure 5B). As mentioned above, efferocytosis at 4 hours reflects mostly primary efferocytosis. Thus, the finding that the values for control and DNMT3A-KO were similar in Figure 5B is consistent with our in vitro data showing intact primary efferocytosis in DNMT3A-KO macrophages (Supplemental Figure 1A). We then analyzed all 4 groups (PBS- or DEX-treated control and H-DNMT3A–KO mice) for nuclear 8-OHdG in thymic macrophages. Macrophage nuclear 8-OHdG levels were lower in the thymi of DEX- versus PBS-injected control mice, which correlated with increased efferocytosis by thymic macrophages in the DEX cohort, but nuclear 8-OHdG levels in thymic macrophages were not lower in DEX- versus PBS-injected H-DNMT3A–KO mice (Figure 5C). Moreover, nuclear PARP1 and MTH1 were increased in thymic macrophages of DEX- versus PBS-injected control mice but not in DEX- versus PBS-injected KO mice (Figure 5, D and E). Importantly, when DEX-treated WT mice were injected with PJ34 to inhibit PARP1 or with TH287 to inhibit MTH1, nuclear 8-OHdG levels were elevated in thymic macrophages compared with mice injected with vehicle control (DMSO) (Figure 5F). As with our in vitro data, this finding was not due to inhibition of primary efferocytosis by these inhibitors (Supplemental Figure 5B). These data provide strong evidence that the DNMT3A/PARP1/MTH1 pathway of efferocytosis-induced oxDNA suppression occurs in vivo.

A major goal in this experiment was to determine if EIMP in thymic macrophages, which is stimulated by DEX treatment in WT mice at 18 hours as a result of the increase in apoptotic thymocytes (7, 15), was blunted in DEX-treated H-DNMT3A–KO mice. We have shown previously that H-DNMT3A–KO in DEX-treated mice compromises continuing efferocytosis in thymic macrophages, as assayed by quantifying the ratio of macrophage-associated versus free TUNEL^+^ cells (8). H-DNMT3A KO also causes thymic necrosis, identified by regions of hypocellularity and/or small, fragmented nuclei (8). Here, we verified both of these findings in this experiment (Supplemental Figure 5, C and D). We now show that EIMP, which we assayed by quantifying the percentage of thymic macrophages expressing Ki67 (7, 15), was markedly lower in the thymi of DEX-treated H-DNMT3A–KO mice than in control mice (Figure 5G). Moreover, treatment with the MTH1 inhibitor TH287 blocked continuing efferocytosis and EIMP in the thymi of DEX-treated WT mice (Figure 5, H and I), accompanied by increased thymic necrosis (Figure 5J). Note the total number of thymic macrophages was not affected by either DNMT3A-KO or TH287 despite lower EIMP (Supplemental Figure 5, E and F), which is consistent with the concept that EIMP increases the relative proportion of pro-resolving macrophages in tissues rather than the total number of macrophages (7). In sum, these data link the DNMT3A/PARP1/MTH1 oxDNA repair pathway during efferocytosis to EIMP and tissue resolution in vivo.

### Evidence of impaired efferocytosis-induced suppression of oxDNA in atherosclerosis regression in a model of DNMT3A CH.

DNMT3A LoF variants are a common cause of CH, which is a risk factor for atherosclerotic cardiovascular disease (CVD) (29–38). We therefore investigated whether a CVD-relevant DNMT3A-CH mutation, R882H (R878H in mice) (35, 57), impairs efferocytosis-induced suppression of oxDNA and EIMP in an atherosclerosis setting in mice. We used an R878H/WT chimeric bone marrow transplantation protocol to reflect the chimeric nature of hematopoietic CH mutations in humans. As a human-relevant endpoint, we chose to examine lesional fibrous cap thickness in response to LDL lowering (“atherosclerosis regression”). In humans, marked LDL lowering promotes fibrous cap thickening, which is a marker of low-risk, stable plaques (58) and of a lower risk of CVD events (24–27). Importantly, activation of efferocytosis-resolution pathways in regressing lesional macrophages, including EIMP, plays a key role in cap thickening after LDL lowering (6, 7, 9, 28).

Accordingly, one group of irradiated *Ldlr^–/–^* mice, referred to as chimeric RH (R878H), were transplanted with 20% bone marrow cells from GFP^–^ *Dnmt3a^R878H^Csf1rCre^Esr1+^* mice (tamoxifen-inducible DNMT3A-RH) and 80% bone marrow cells from GFP^+^ C57BL/6J WT mice. Another group of mice, referred to as chimeric WT, were transplanted with 20% bone marrow cells from GFP^–^ *Csf1rCre^Esr1+^* mice (DNMT3A-WT) and 80% bone marrow cells from GFP^+^ C57BL/6J WT mice. After 4 weeks of recovery, all mice were treated with tamoxifen to promote expression of *Dnmt3a^R878H^* in the *Dnmt3a^R878H^Csf1rCre^Esr1+^* cells in the chimeric RH cohort. The mice were then fed a Western diet for 17 weeks. Some of the mice were harvested (baseline), while others were subjected to LDL lowering for 6 weeks by switching to a chow diet and injecting them with an antisense oligonucleotide targeting apolipoprotein B100 (apoB ASO) to induce regression (28) (Supplemental Figure 6A). As designed, the combination of a chow diet and apoB ASO markedly lowered plasma cholesterol, and this effect was similar in the 2 cohorts (Supplemental Figure 6B). Mice in the regression cohorts had lower body weights, as expected (6, 7, 9), but body weights were similar among the chimeric RH and WT mice (Supplemental Figure 6C). Peripheral blood cell counts were similar across all cohorts except for a modest lowering of WBC numbers in chimeric RH versus WT regression mice (Supplemental Figure 6D). There was only a modest increase in the percentage of GFP^–^ RH cells in the chimeric RH mice as compared with GFP^–^ WT cells in the chimeric WT mice (Supplemental Figure 6E), which is consistent with a less pronounced expansion of peripheral WBCs in humans with DNMT3A-CH compared with other CH variants (59).

Lesional analysis revealed that nuclear 8-OHdG in lesional macrophages was lower in regressing versus baseline lesions in the WT cohort (Figure 6A, group 3 vs. group 1), consistent with increased macrophage efferocytosis in regression and our finding here that efferocytosis lowered oxDNA (6, 7, 9, 28). In contrast, macrophage nuclear 8-OHdG levels were not decreased in the regressing lesions of chimeric RH mice (Figure 6A, group 4 vs. group 2). Nuclear 8-OHdG levels in non-Mac2 regions did not differ significantly between WT and RH regression lesions (Supplemental Figure 6F), supporting a predominantly macrophage-associated effect. Consistent with our findings related to the DNA repair pathway shown in vitro and in the DEX thymus model, nuclear PARP1 and MTH1 in lesional macrophages were elevated in regressing versus baseline WT lesions but not RH lesions (Figure 6, B and C). With regard to EIMP, whereas total lesional macrophages decreased in regression as shown previously (60) and here (Supplemental Figure 6G), the pro-resolving efferocytic macrophage subpopulation increased as a result of EIMP (7). We now demonstrate that this increase was blunted in chimeric RH regressing lesions (Figure 6D). In 6-week regressing lesions, the ratio of macrophage-associated versus free TUNEL^+^ cells largely reflects continuing efferocytosis (6–9, 14), and we found that this pro-resolving endpoint was markedly decreased in chimeric RH versus WT regressing lesions (Figure 6E).

To further assess the presence of pro-resolving macrophage features during regression, we examined the expression of the pro-resolving markers TGF-β1 and arginase 1 in plaque macrophages. In chimeric WT mice, both markers were increased during regression compared with baseline. In contrast, TGF-β1 was not increased during regression in chimeric RH mice, whereas arginase 1 was increased in chimeric RH regression but not to the level seen in chimeric WT regressing lesions (Supplemental Figure 7, A and B). These data are consistent with our previous finding that EIMP expands the pool of macrophages that are particularly adept at carrying out continuing efferocytosis (7). Another marker of a pro-resolving environment is an increase in FoxP3^+^ Tregs (61), which can interact with macrophages to promote efferocytosis, including in the setting of atherosclerosis regression (28, 62). In this context, the FoxP3^+^/CD3^+^ T cell ratio was increased in regressing chimeric WT lesions versus baseline, whereas this increase was not observed in chimeric RH lesions (Supplemental Figure 7C).

Finally, the most important human-relevant endpoint of LDL-lowering–induced atherosclerosis regression is fibrous cap thickening. We found that regression-induced cap thickening, clearly evident in the WT cohort, was markedly abrogated in the chimeric RH cohort (Figure 6F). The effect of chimeric RH in cap thickness in regression could not be explained by overall changes in the non-cap lesion area or the necrotic area, as although both parameters showed modest trends toward lower values in regression versus baseline, there were no differences between WT and chimeric mice in these endpoints at either baseline or during regression (Supplemental Figure 7, D and E). Moreover, cap-localized lumican^+^ cells, which are collagen-producing cells that contribute to fibrous cap formation (63), were increased in chimeric WT regression lesions compared with baseline, and this increase was blunted in chimeric RH mice (Supplemental Figure 7C). Thus, in an experimental model of DNMT3A-CH, the PARP1/MTH1 oxDNA repair pathway and its pro-resolving effects, notably EIMP, were impaired in the setting of LDL-lowering–induced plaque stabilization.

Although comparing regressing versus progressing lesions from humans is not feasible because of the lack of availability of plaque material obtained after LDL lowering, we were able to compare stable versus unstable regions of individual human carotid plaques obtained from 10 individuals who underwent clinically indicated carotid endarterectomies. As we published previously, the unstable regions of plaques have more lesional necrosis, less fibrosis, and a lower content of pro-resolving, pro-efferocytic mediators than the stable regions (64). Here, we show that lesional macrophages in the unstable regions had, on average, higher nuclear 8-OHdG and lower nuclear PARP1 and MTH1 than did macrophages in stable regions (Figure 6G and Supplemental Figure 8, A and B). Notably, linear regression analysis revealed significant inverse correlations between nuclear 8-OHdG and both PARP1 and MTH1 levels in carotid plaque macrophages (Figure 6H). These data show an association between unstable plaques and decreased biochemical markers of the PARP1/MTH1 oxDNA repair pathway in humans.

## Discussion

Efferocytosis reprograms macrophages to be pro-resolving through a variety of upstream pathways triggered by efferocytosis receptor signaling, changes in macrophage energy metabolism, and/or molecules derived from the phagolysosomal degradation of ACs, including methionine and nucleotides (6–9, 12–15). These pathways induce downstream pro-resolving processes, including IL-10 and TGF-β1 production, continuing efferocytosis, and pro-resolving macrophage proliferation (EIMP) (6–9, 12–15). In previous studies, links between the upstream triggers and the downstream resolution endpoints have involved cytoplasmic pathways that alter gene expression, posttranslational protein modification, or cytoskeletal remodeling. Here, we introduce what we believe to be a new mechanism involving DNA repair, which links an upstream AC-derived molecule (methionine) to a key resolution endpoint (EIMP).

EIMP requires multiple inputs to ensure the activation of cell proliferation only in the setting of abundant ACs (7, 15–22). Previously described efferocytic inputs required for EIMP include receptor-mediated signaling through the efferocytosis receptor MerTK, nucleotides released from AC phagolysosomal degradation (7), and lactate released by enhanced macrophage glycolysis supporting MYC protein stabilization (15). The work here introduces the suppression of oxDNA as a prerequisite checkpoint for EIMP. Moreover, our study reveals that the AC-methionine/DNMT3A pathway initiates more than 1 pro-resolving action, as we showed previously that it represses the expression of the ERK phosphatase DUSP4, enabling the expression of 2 pro-resolving mediators, prostaglandin E_2_ and TGF-β1 (8).

The finding that efferocytosis-induced suppression of oxDNA requires DNMT3A has potentially important implications, as *DNMT3A* is the most common gene mutated in CH (65, 66). On the basis of findings of this study and of previous studies (7, 8, 28, 62), we propose that the failure of DNMT3A-mediated oxDNA suppression, which leads to defects in EIMP and is associated with lower levels of Arg1^+^ macrophages, TGF-β1, and Tregs, contributes to disease processes linked to DNMT3A-CH, notably atherosclerosis (29–38). Other diseases associated with DNMT3A-CH that, in theory, could also involve this mechanism include heart failure (67) and aortic valve stenosis (68), both of which are associated with changes in Tregs (68, 69), as well as with osteoporosis (70), neurodegenerative disease (71, 72), metabolic disease (73), and nonhematologic malignancies (74). Given that DNMT3A-CH is a disease of aging (75) and that BER function declines with age (76), the defect in BER-mediated suppression of oxDNA in efferocytosing macrophages may be particularly pronounced in the aged population with DNMT3A-CH. Impaired tissue resolution is likely additive with other pathogenic consequences of DNMT3A-CH. For example, previous work unrelated to efferocytosis/resolution has shown that loss of DNMT3A function in macrophages activates pro-inflammatory pathways that accelerate osteoporosis and atherosclerosis progression (70, 77). In the osteoporosis study (70), impaired DNA methylation in DNMT3A-deficient macrophages was linked to increased binding of the proinflammatory transcription factors IRF3 and RelA to DNA. The atherosclerosis study (77) also suggested an inflammatory signature in DNMT3A-mutant macrophages. In particular, atherosclerotic lesions of *Ldlr^–/–^* mice transplanted with chimeric DNMT3A-KO bone marrow and fed a high-fat, high-cholesterol diet revealed a distinct adventitial macrophage population that had features of tissue-resident macrophages but with an inflammatory cytokine profile (52).

For our chimeric DNMT3A-CH experiment, we used bone marrow cells harboring the human-like R878H mutation (R882H in humans), which is a potent LoF CH mutation in the methyltransferase domain (35, 57). The use of this particular mutant is important for 2 reasons. First, although DNMT3A can have noncanonical functions in cells that do not involve DNA methylation (43), the DNMT3A pathways that macrophages use to link efferocytosis to resolution involve DNA methylation (ref. 8 and the present study), which highlights the importance of the methyltransferase domain of DNMT3A in these processes. Second and most important, evidence is emerging that individuals with DNAMT3A-CH, who have this mutation, which is in the methylase domain and acts in a dominant-negative manner (78, 79), have the highest risk of CVD (35, 38, 40). Indeed, while there are some studies in humans that have failed to show a link between DNMT3A-CH and CVD, the cohorts in these studies may have included a large number of people with other types of DNMT3A-CH mutations, particularly in non-methyltransferase regions of DNMT3A (35). Moreover, an important recent study has raised the possibility that a lack of deep sequencing in previous DNMT3A-CH cardiovascular studies could have led to false-negative results (80).

Our area of focus in atherosclerosis has been on the role of pro-resolving efferocytic macrophages in promoting fibrous cap thickening (6–9), a key tissue resolution process strongly associated with a decreased risk of acute atherosclerotic cardiovascular events in humans (81). In this study, we examined a critical, clinically relevant process known as “atherosclerosis regression,” in which plaques develop thicker fibrous caps in response to marked LDL lowering (24–27). We and others have shown previously that fibrous cap thickening in regression requires efferocytosis-induced tissue resolution (2, 6, 7, 9, 23, 28, 82). Here, we discovered that the regressing lesions of chimeric DNMT3A-CH mice had thinner fibrous caps and decreased numbers of cap-localized lumican^+^ cells, which contribute to cap formation (63), along with evidence of impaired PARP1/MTH1-mediated oxDNA repair and defective EIMP. EIMP leads to an increase in the number of pro-resolving macrophages that can make TGF-β1 (7), and the aforementioned DNMT3A/DUSP4/ERK pathway leads directly to an increase in the synthesis and secretion of TGF-β1 in macrophages (8). As TGF-β1 is a pro-fibrotic factor involved in fibrous cap thickening (83), these 2 DNMT3A pathways provide a possible mechanistic link between loss of DNMT3A function in CH and the development of unstable human plaques. Indeed, we show here that thin-capped, unstable human plaques have higher levels of 8-OHdG and lower nuclear PARP1 and MTH1 levels in lesional macrophages compared with more stable lesions. Moreover, linear regression analysis revealed significant inverse correlations between nuclear 8-OHdG and both PARP1 (*P* value 0.018) and MTH1 (*P* value 0.0354) in carotid plaque macrophages. Interestingly, previous work has shown that urinary 8-OHdG levels are associated with cardiovascular events in humans (84, 85). Moving forward, we aim to compare features of plaque stability in atheroma specimens from people with and without DNMT3A-CH methyltransferase mutations. In particular, we predict that the lesions of individuals with DNMT3A-CH, particularly those with mutations in methyltransferase domain (see above), will have higher lesional oxDNA, impaired proliferation of pro-resolving macrophages, and thinner fibrous caps. Another future goal will be to integrate our findings related to atherosclerosis fibrous cap thinning in DNMT3A-CH with other settings in which DNMT3A-CH affects tissue fibrosis. For example, a previous study showed a link between DNMT3A-CH and pathological fibrosis in the heart, with a mechanism related to cardiac fibroblast activation (69). Thus, it will be interesting to understand how DNMT3A-CH prevents beneficial activation of fibroblast-like cells in atherosclerosis while enhancing pathologic cardiac fibroblast activation in fibrotic heart disease.

Another future goal is to determine the precise molecular mechanism linking DNMT3A-mediated DNA methylation to the PARP1/MTH1 oxDNA repair pathway. Our data suggest that the key downstream event is chromatin binding of PARP1 and nuclear localization of MTH1. One possibility is that the methylation at specific genomic regions, perhaps by altering gene expression, activates one or more processes that lead to the nuclear recruitment of DNA repair enzymes. However, the rapid suppression of 8-OHdG following AC uptake may point to a nontranscriptional mechanism, albeit one that is still dependent on DNA methylation. For example, DNMT3A-mediated DNA methylation could alter overall chromatin architecture (86) in a way that promotes the rapid chromatin binding of PARP1 after efferocytosis. Additional future goals include understanding how the repair and hydrolysis of 8-OHdG license macrophages for proliferation after efferocytosis and, conversely, identifying the mechanism of cell-cycle blockade when oxDNA repair is impaired due to DNMT3A LoF. Moreover, we are interested in exploring other consequences of DNMT3A-dependent PARP1 chromatin binding in efferocytosing macrophages, e.g., in the regulation of gene expression (87). Finally, future studies using temporal macrophage *Mth1* deletion in our preclinical mouse models will further define the DNMT3A-mediated BER pathway in vivo and help define potential therapeutic strategies for DNMT3A-CH diseases.

In summary, this study uncovers an efferocytosis-induced tissue resolution mechanism involving activation of a BER-mediated oxDNA repair pathway. We propose that this pathway could be highly relevant to the hundreds of millions of people on LDL-lowering medications, as such therapy promotes plaque stabilization through mechanisms linked to efferocytosis-induced resolution (6, 7, 9, 28). The finding that the oxDNA repair pathway requires DNMT3A-mediated DNA methylation provides a mechanistic link between DNMT3A-CH and atherosclerotic CVD. Thus, a key question moving forward is whether humans with methyltransferase-defective DNMT3A-CH have impaired regression in response to lowered LDL and whether this response is due to defective efferocytosis resolution pathways in lesional macrophages, including suppression of oxDNA and impaired EIMP. The answers to these questions have potentially impactful therapeutic implications, given the growth of preclinical work examining ways to boost efferocytosis and resolution for atherosclerosis and other chronic inflammatory diseases driven by impaired efferocytosis and tissue resolution (88–94).

## Methods

### Sex as a biological variable.

The in vivo experiments in this study involved the use of male mice. Most of the in vitro studies used BMDMs from male mice, but the efferocytosis-induced 8-OHdG suppression finding was confirmed in BMDMs from female mice. HMDMs studies were conducted using blood monocytes from anonymous healthy adult donors whose sex was unidentified (New York Blood Center). Human carotid endarterectomy specimens were from both female (21%) and male (79%) individuals. Although sex was not analyzed as an independent variable, we expect that the key findings in this study will be relevant in both males and females.

### Statistics.

GraphPad Prism 10 (GraphPad Software) was used for all statistical analyses. Data were tested for normality using either the D’Agostino-Pearson or the Shapiro-Wilk test. Data that passed the normality test were analyzed using the 2-tailed Student’s *t* test for comparison of 2 groups or 1-way ANOVA with Fisher’s least-significant difference (LSD) post hoc analysis for comparison of more than 2 groups. Data that were not normally distributed were analyzed using the Mann-Whitney *U* test or the Kruskal-Wallis test. Data are shown as mean values ± SEM, and differences were considered statistically significant at a *P* value of less than 0.05. The *N* number for in vitro experiments refers to the number of wells of macrophages, and the *n* number for the mouse experiments refers to the number of mice. For normalization of data, all values were divided by the means of the control group. No data were excluded.

### Study approval.

The Columbia University’s IRB and Health Insurance Portability and Accountability Act guidelines were followed for isolating peripheral human blood leukocytes. Mouse protocols were approved by Columbia University’s IACUC. All mice were cared for according to the NIH guidelines for the care and use of laboratory animals, and all were in good general health based on appearance and activity. The use of deidentified carotid artery specimens, which were removed for clinical indications only and would otherwise have been discarded, conformed with the declaration of Helsinki and were approved for use by the Johannes Gutenberg University ethics review board. Informed written consent was obtained from each patient. The Columbia University IRB provided ethics approval for these studies, and procedures were conducted in accordance with an approved IRB protocol.

### Data availability.

Values for all data points in the graphs are reported in the Supporting Data Values file.

Additional detailed methods can be found in the Supplemental Methods.

## Author contributions

KA and IT conceived and designed the research. KA conducted the experiments and collected and analyzed the data. DN, SRS, PBA, JR, HZ, JG, and ADV provided critical advice during the course of the project. BD donated patient samples, and BD, HW, and LM were instrumental in the interpretation of the human data. KA and IT wrote the manuscript, with comments provided by all other authors.

## Conflict of interest

The authors have declared that no conflict of interest exists.

## Funding support

This work is the result of NIH funding, in whole or in part, and is subject to the NIH Public Access Policy. Through acceptance of this federal funding, the NIH has been given a right to make the work publicly available in PubMed Central. The content of this work is solely the responsibility of the authors and does not necessarily represent the official views of the NIH.

- NIH grant R35-HL145228 (to IT).
- NIH grant K99-HL181285 (to SRS).
- NIH grant P01-HL172741 (to IT).
- American Heart Association Postdoctoral Fellowship Award (to DN).
- Translational Immunology Core Facility NIH grants P30CA013696, S10RR027050, and S10OD020056.
- Molecular Pathology Shared Resource of the Herbert Irving Comprehensive Cancer Center at Columbia University grant P30CA013696.

## Supplementary Material

Supplemental data

Unedited blot and gel images

Supporting data values

## Figures and Tables

**Figure 1:**
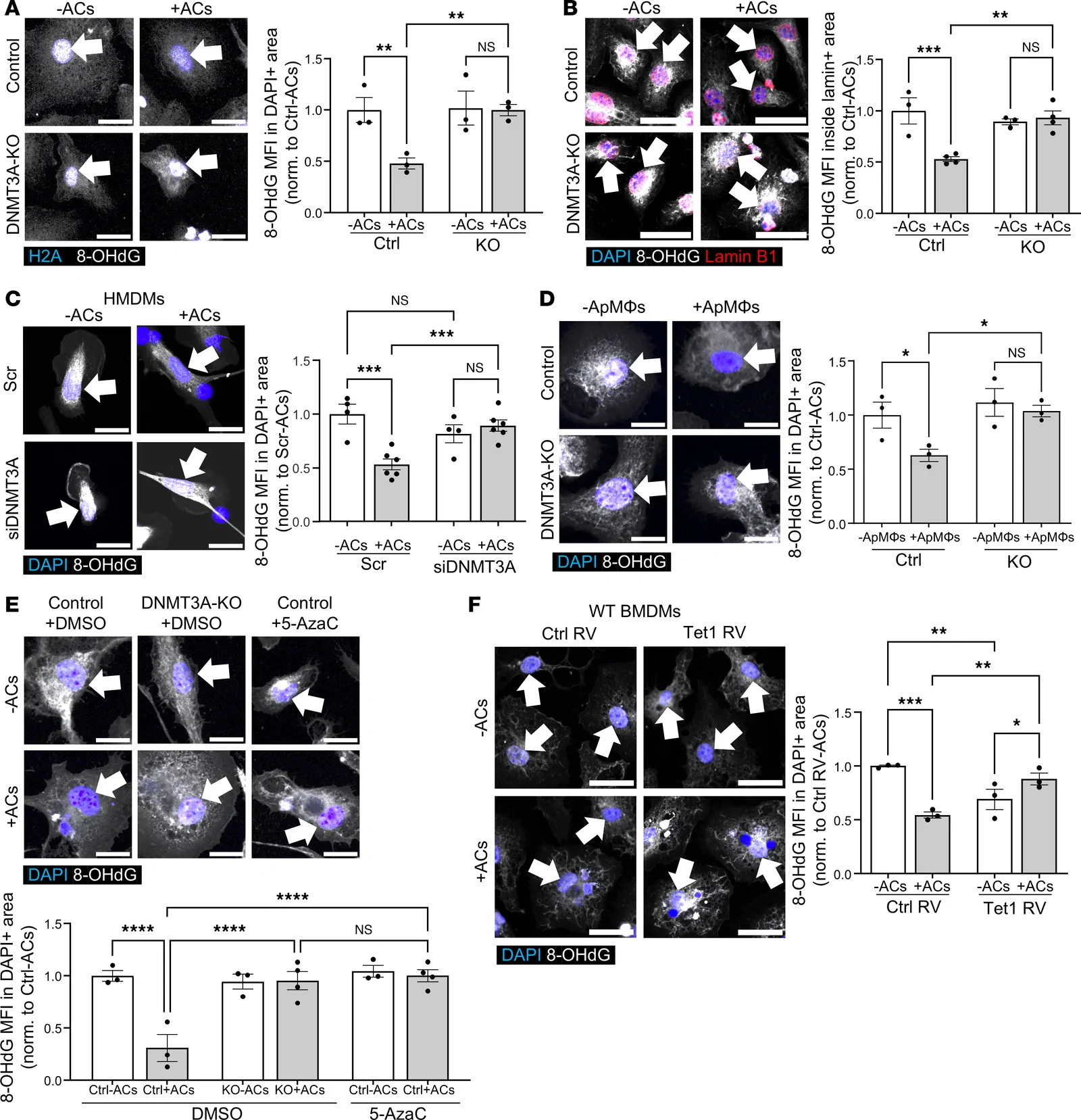
Efferocytosis induces suppression of oxDNA in a DNMT3A-dependent manner. (**A**) BMDMs from WT (Ctrl) or *Dnmt3a^–/–^* (KO) mice were incubated without or with ACs (–ACs, +ACs) for 45 minutes, after which noninternalized ACs were removed by rinsing. After an additional 1-hour incubation, the cells were immunostained for 8-OHdG and H2A (nucleus), and 8-OHdG MFI in H2A^+^ areas (arrows) were quantified by confocal fluorescence microscopy (*n* = 3 replicates/group; similar results were observed in 3 replicate experiments). (**B**) Similar to **A**, but BMDMs were immunostained for the nuclear envelope marker lamin B1, and 8-OHdG MFI inside lamin B–stained nuclei (arrows) was quantified (*n* = 3–4 replicates/group). (**C**) Similar to **A**, but HMDMs were treated with scrambled RNA (Scr) or siDNMT3A, and 8-OHdG MFI in DAPI^+^ nuclei (arrows) was quantified (*n* = 4–6 replicates/group). (**D**) Similar to **A**, but BMDMs were exposed to apoptotic HMDMs and quantified for 8-OHdG MFI in DAPI^+^ nuclei (arrows) (*n* = 3 replicates/group). MΦs, macrophages. (**E**) Control or DNMT3A-KO BMDMs were pretreated for 2 hours with vehicle (DMSO) or 20 μM 5-azacytidine (5-AzaC) and then quantified for 8-OHdG MFI in DAPI^+^ areas (arrows) (*n* = 3–4 replicates/group). (**F**) Mouse bone marrow stem cells were transduced with control (Ctrl) or Tet1 retrovirus (Tet1 RV) and then incubated for 7 days with macrophage-differentiation medium containing macrophage colony-stimulating factor (M-SCF). The resulting macrophages were quantified for 8-OHdG MFI in DAPI^+^ nuclei (*n* = 3 replicates/group). Arrows indicate nuclei. Scale bars: 50 μm. Data indicate the mean ± SEM. *P* values were determined by 2-way ANOVA (**A**–**D**, and **F**) and 1-way ANOVA (**E**) with Fisher’s LSD post hoc analysis. *P < 0.05, **P < 0.01, ***P < 0.001, and ****P < 0.0001. norm., normalized.

**Figure 2:**
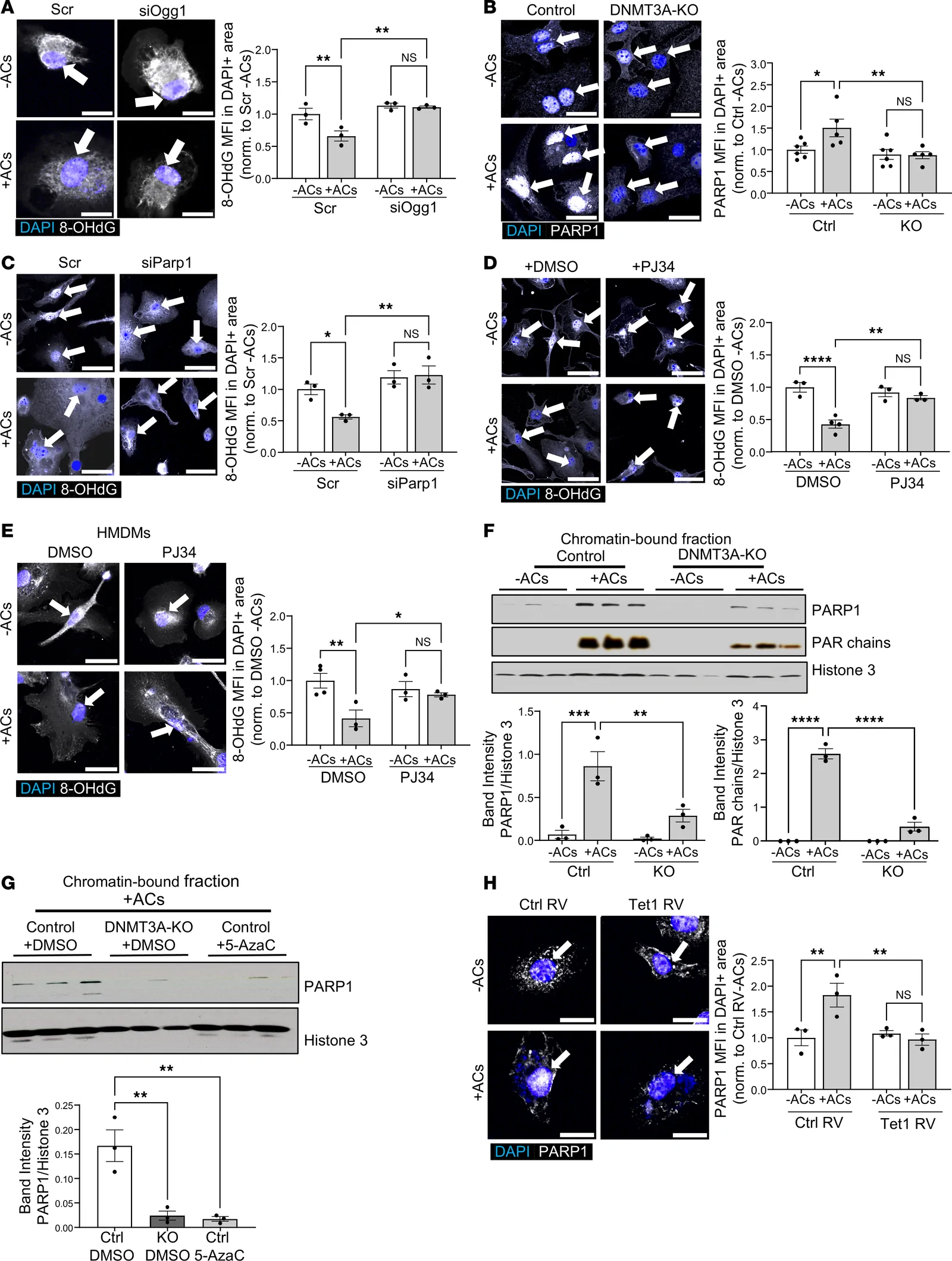
Efferocytosis-induced suppression of oxDNA is dependent on PARP1. (**A**) BMDMs pretreated with scrambled (Scr) or siOgg1 were incubated with or without ACs for 45 minutes, rinsed, and then incubated for an additional hour. The cells were immunostained for 8-OHdG and quantified for 8-OHdG MFI in DAPI^+^ areas (*n* = 3 replicates/group). Arrows point to nuclei. (**B**) BMDMs were incubated with or without ACs for 45 minutes, rinsed, and then incubated for an additional hour. The cells were immunostained for PARP1 and quantified for PARP1 MFI in DAPI^+^ areas (*n* = 6 replicates/group). Arrows indicate nuclei. (**C**) As in **A**, but the BMDMs were pretreated with scrambled or siParp1. (**D**) As in **A**, but the BMDMs were pretreated for 2 hours with vehicle (DMSO) or 20 μM PJ34. (**E**) As in **D**, except HMDMs were used. (**F**) Control or DNMT3A-KO BMDMs were incubated with or without ACs for 45 minutes, rinsed, and then incubated for an additional hour. Chromatin fractions were immunoblotted for PARP1, poly-mono-ADP ribose, and histone 3 and quantified for the density ratio of PARP1/histone 3 and PARylated protein/histone 3 (*n* = 3 replicates/group). (**G**) WT (control) or DNMT3A-KO BMDMs were pretreated for 2 hours with vehicle (DMSO) or 20 μM 5-AzaC and then incubated with or without ACs as in **F**. Chromatin fractions were immunoblotted for PARP1 and histone 3 and quantified for the PARP1/histone 3 ratio (*n* = 3 replicates/group). (**H**) Macrophages transduced with control or Tet1 RV (Ctrl or Tet1 RV) were incubated with or without ACs as in Figure 1F and quantified for PARP1 in DAPI^+^ areas (*n* = 3 replicates/group). Scale bars: 50 μm. Data show the mean ± SEM. *P* values were determined by 2-way ANOVA (**A**–**F**, and **H**) or 1-way ANOVA (**G**) with Fisher’s LSD post hoc analysis. *P < 0.05, **P < 0.01, ***P < 0.001, and ****P < 0.0001.

**Figure 3:**
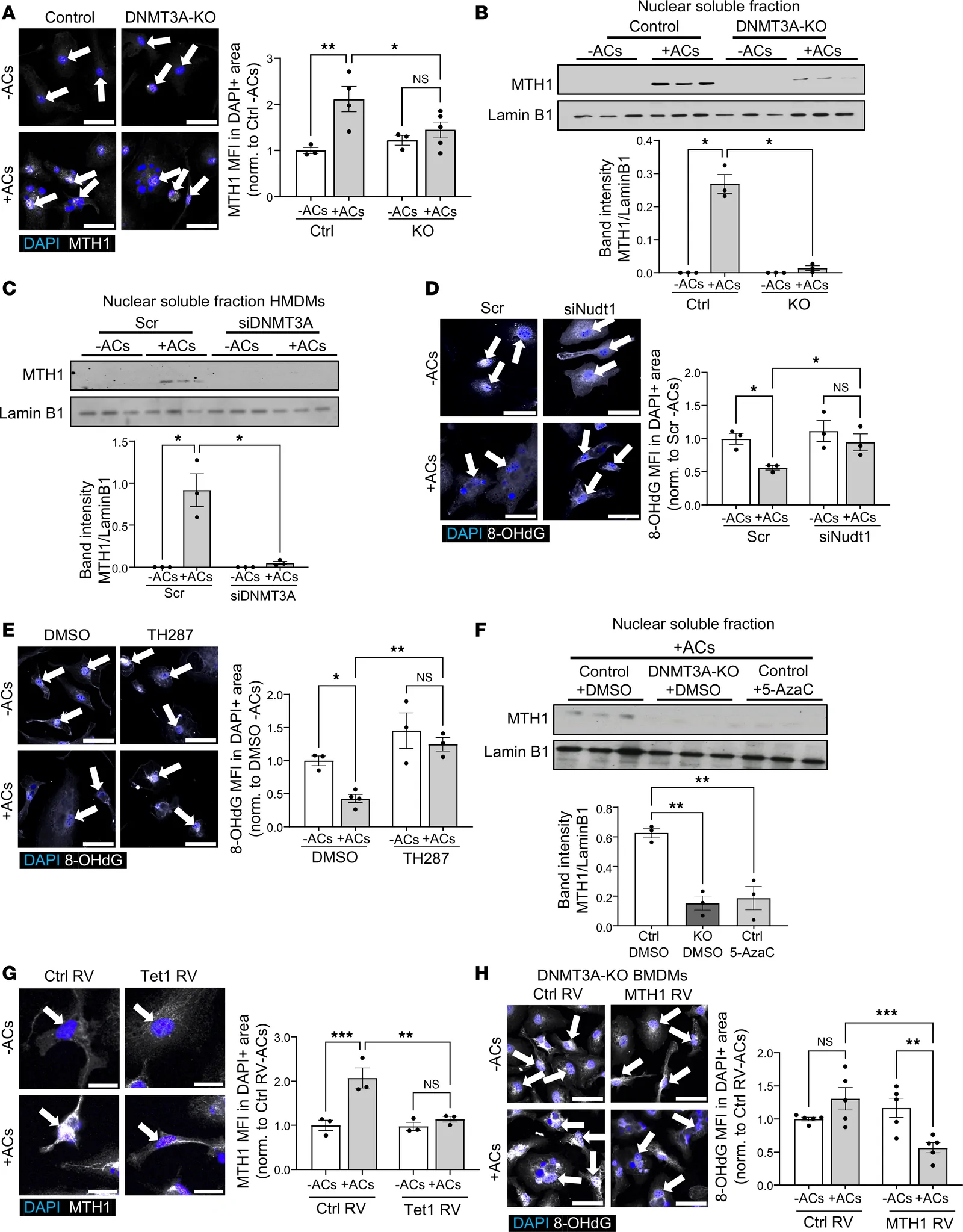
Efferocytosis-induced suppression of oxDNA is dependent on MTH1. (**A**) BMDMs were incubated with or without ACs for 45 minutes, rinsed, and then incubated for an additional hour. Cells were immunostained for MTH1 and stained with DAPI (nuclei) and then quantified for MTH1 MFI in DAPI^+^ areas (*n* = 3–5 replicates/group). Arrows indicate nuclei. (**B**) Control or DNMT3A-KO BMDMs were incubated with or without ACs for 45 minutes, rinsed, and then incubated for an additional hour. The nuclear soluble fraction was immunoblotted for MTH1 and lamin B1 and quantified for the MTH1/lamin B1 ratio (*n* = 3 replicates/group). (**C**) Similar to **B**, but with HMDMs (*n* = 3 replicates/group). (**D**) As in **A**, but the macrophages were pretreated with scrambled or si*Nudt1* (gene for MTH1), immunostained for 8-OHdG, and quantified for 8-OHdG MFI in DAPI^+^ areas (*n* = 3 replicates/group). Arrows indicate nuclei. (**E**) Similar to **D**, except the cells were pretreated for 2 hours with vehicle (DMSO) or 20 μM TH287. Cells were quantified for 8-OHdG MFI in DAPI^+^ areas (arrows) (*n* = 3 replicates/group). (**F**) As in **B**, but the control and DNMT3A-KO BMDMs were pretreated for 2 hours with vehicle (DMSO) or 20 μM 5-AzaC. The nuclear soluble fraction was immunoblotted for MTH1 and lamin B1 and quantified for the ratio of MTH1/lamin B1 (*n* = 3 replicates/group). (**G**) Macrophages transduced with control or Tet 1 RV were prepared as in Figure 1F and quantified for MTH1 MFI in DAPI^+^ area (*n* = 3 replicates/group). (**H**) As in **D**, except that the DNMT3A-KO macrophages were transduced with control or MTH1 RV (Ctrl or MTH1 RV). Cells were quantified for 8-OHdG in DAPI^+^ areas (arrows) (*n* = 5 replicates/group). Scale bars: 50 μm. Data indicate the mean ± SEM. *P* values were determined by 2-way ANOVA (**A**–**E**, **G**, and **H**) or 1-way ANOVA (**F**), with Fisher’s LSD post hoc analysis. *P < 0.05, **P < 0.01, and ***P < 0.001.

**Figure 4:**
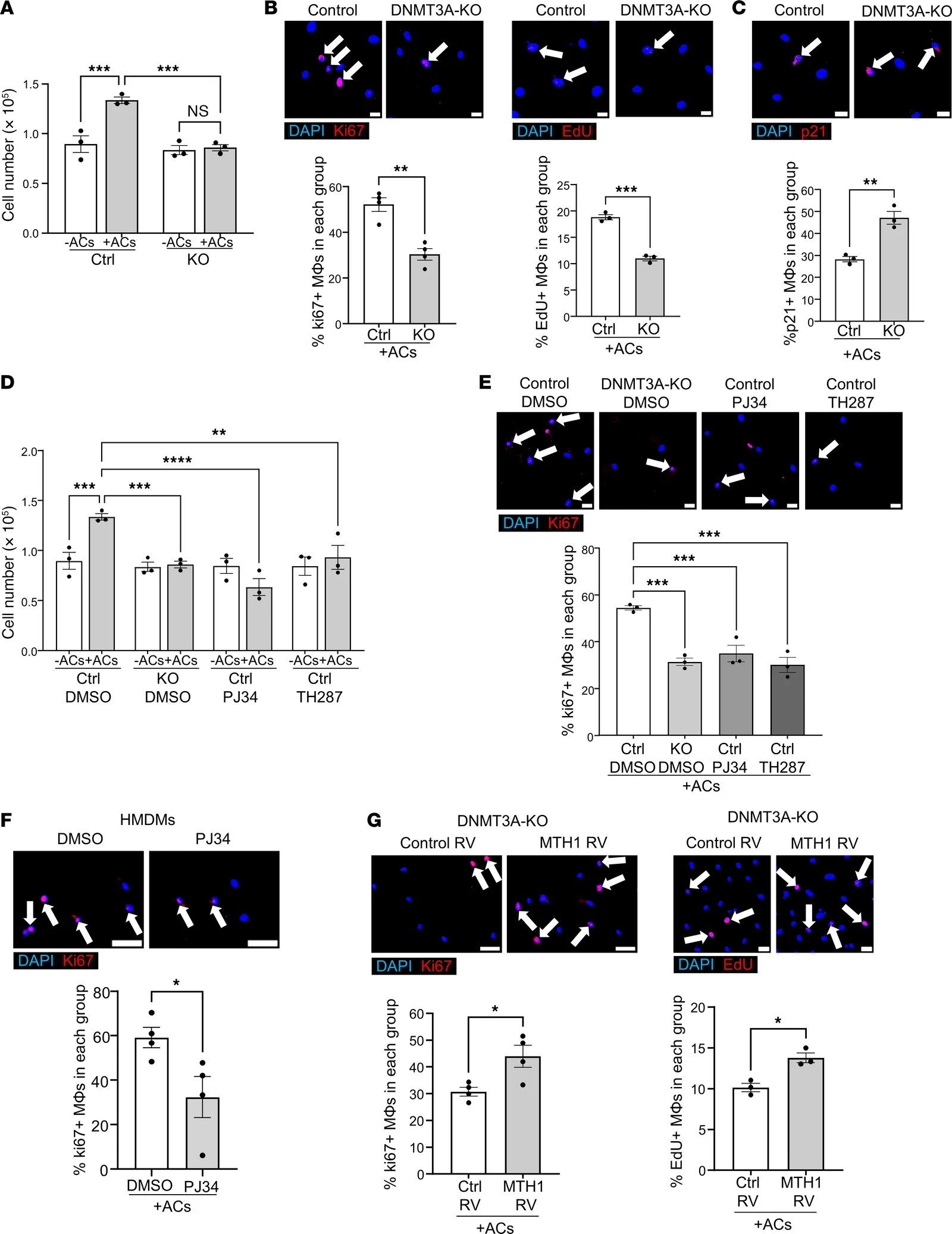
Efferocytosis-induced suppression of oxDNA is required for EIMP. (**A**) BMDMs from control or DNMT3A-KO mice were incubated with or without ACs for 45 minutes, rinsed, and then incubated for an additional 24 hours. Cell numbers were quantified (*n* = 3 replicates/group). (**B**) Similar to **A**, but the percentage of Ki67^+^ macrophages or EdU^+^ macrophages (after a 2-hour pretreatment with 20 μM EdU) was quantified using immunofluorescence microscopy (*n* = 3–4 replicates/group). (**C**) Similar to **B**, but the percentage of p21^+^ macrophages was quantified (*n* = 3 replicates/group). (**D**) Control or DNMT3A-KO BMDMs were pretreated for 2 hours with 20 μM PJ34, TH287, or vehicle control (DMSO). Cells were incubated with or without ACs for 45 minutes, rinsed, and incubated for an additional 24 hours, and then cell numbers were quantified (*n* = 3 replicates/group). (**E**) Similar to **D**, but the percentage of Ki67^+^ macrophages was quantified (*n* = 3 replicates/group). (**F**) HMDMs pretreated with 20 μM PJ34 or vehicle control (DMSO) were incubated with or without ACs for 45 minutes, rinsed, and then incubated for an additional 24 hours. The percentage of Ki67^+^ macrophages was quantified (*n* = 4 replicates/group). (**G**) DNMT3A-KO macrophages were retrovirally transduced with control or MTH1 RV as in Figure 3H. The cells were incubated with or without ACs for 45 minutes, rinsed, and incubated for an additional 24 hours, and the percentage of Ki67^+^ or EdU^+^ macrophages was quantified as in **B** (*n* = 3–4 replicates/group). Scale bars: 50 μm. Data show the mean ± SEM. *P* values were determined by 2-tailed Student’s *t* test (**B**, **C**, **F**, and **G**), 2-way ANOVA (**A** and **D**), and 1-way ANOVA (**E**) with Fisher’s LSD post hoc analysis. *P < 0.05, **P < 0.01, ***P < 0.001, and ****P < 0.0001.

**Figure 5:**
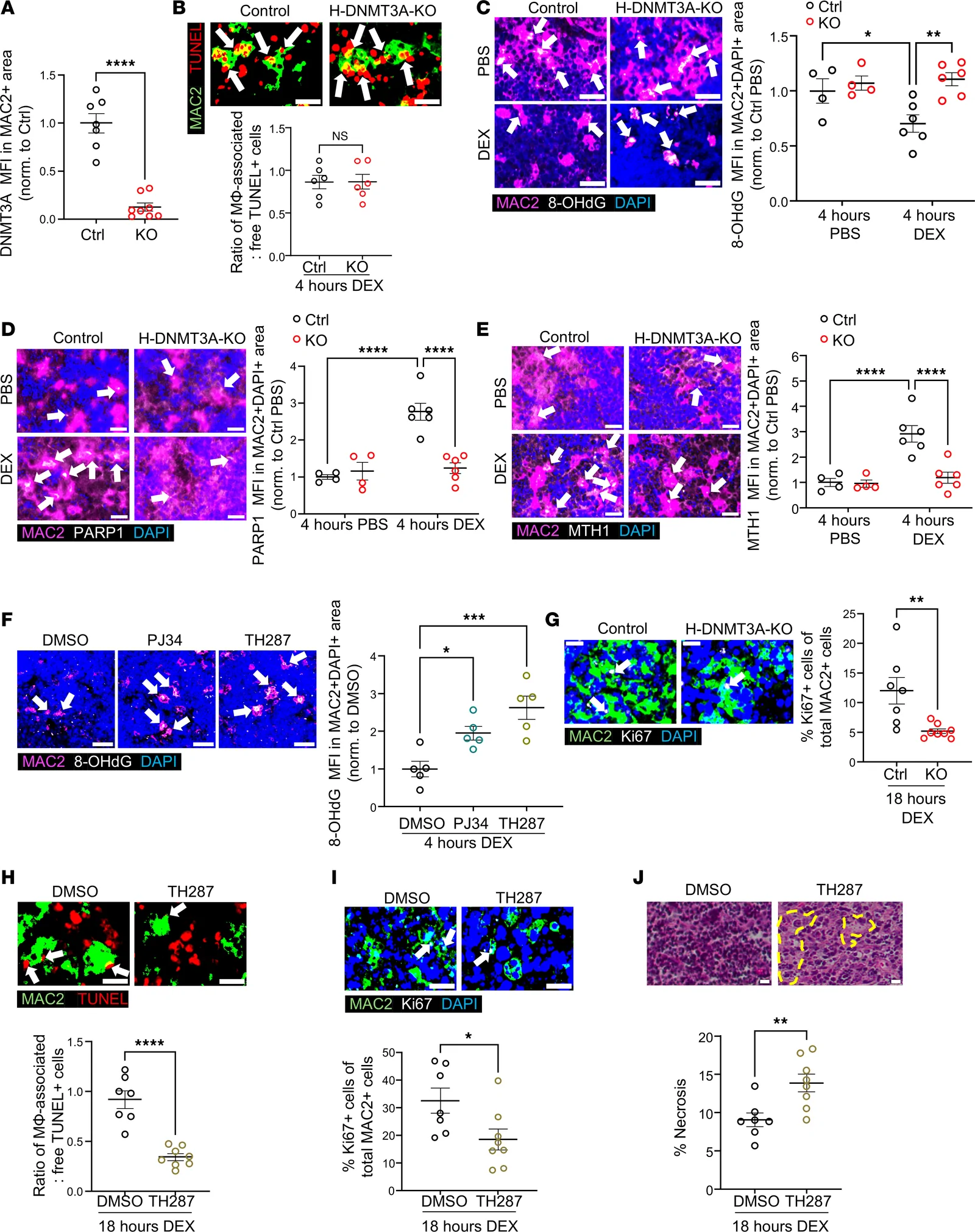
Efferocytosis-induced suppression of oxDNA is required for tissue resolution in vivo. Mice were transplanted with bone marrow from control *Vav1cre^+/–^* (Ctrl) or *Dnmt3a^fl/fl^* *Vav1cre^+/–^* (KO) mice. After 4 weeks, the mice were injected with PBS or DEX, and the thymi were harvested after 4 or 18 hours. The thymic sections harvested at 4 hours were (**A**) immunostained for Mac2 and DNMT3A and quantified for the DNMT3A MFI in Mac2^+^ areas (*n* = 7–8 mice/group); (**B**) quantified for the ratio of macrophage-associated/free TUNEL^+^ cells (arrows) (*n* = 6 mice/group); (**C**) immunostained for Mac2 and 8-OHdG (arrows) and stained with DAPI, with quantification of the 8-OHdG MFI in Mac2^+^DAPI^+^ areas (*n* = 4–6 mice/group); (**D**) immunostained for Mac2 and PARP1 (arrows), stained with DAPI, and quantified as the PARP1 MFI in Mac2^+^DAPI^+^ areas (*n* = 4–6 mice/group); and (**E**) immunostained for Mac2 and MTH1 (arrows) and stained with DAPI, followed by quantification of the MTH1 MFI in Mac2^+^DAPI^+^ areas (*n* = 4–5 mice/group). (**F**) As in **C**, but the mice were treated with 25 mg/kg PJ34, 5 mg/kg TH287, or vehicle (DMSO) 30 minutes before and 15 minutes and 2 hours after DEX (*n* = 5 mice/group). (**G**) Thymi harvested at 18 hours from control or KO mice were immunostained for Mac2 and Ki67 (arrows), stained with DAPI, and quantified to determine the percentage of Ki67^+^ macrophages/thymic macrophages (*n* = 7–8 mice/group). (**H**–**J**) C57BL/6J mice were treated with TH287 as in **F**. After 18 hours, the thymic sections were (**H**) quantified for the ratio of macrophage-associated/free TUNEL^+^ cells (arrows) and (**I**) immunostained for Mac2 and Ki67 (arrows), stained with DAPI, and quantified as the percentage of Ki67^+^ macrophages/thymic macrophages. (**J**) H&E-stained sections were assayed for the percentage of necrotic areas (regions of hypocellularity and/or small, fragmented nuclei). For **H**–**J**, *n* = 7–8 mice/group. Scale bars: 50 μm. Data are shown as the mean ± SEM. *P* values were determined by 2-tailed Student’s *t* test (**A**, **B**, and **G**–**J**), 2-way ANOVA (**C**–**E**), or 1-way ANOVA (**F**), with Fisher’s LSD post hoc analysis. *P < 0.05, **P < 0.01, ***P < 0.001, and ****P < 0.0001.

**Figure 6:**
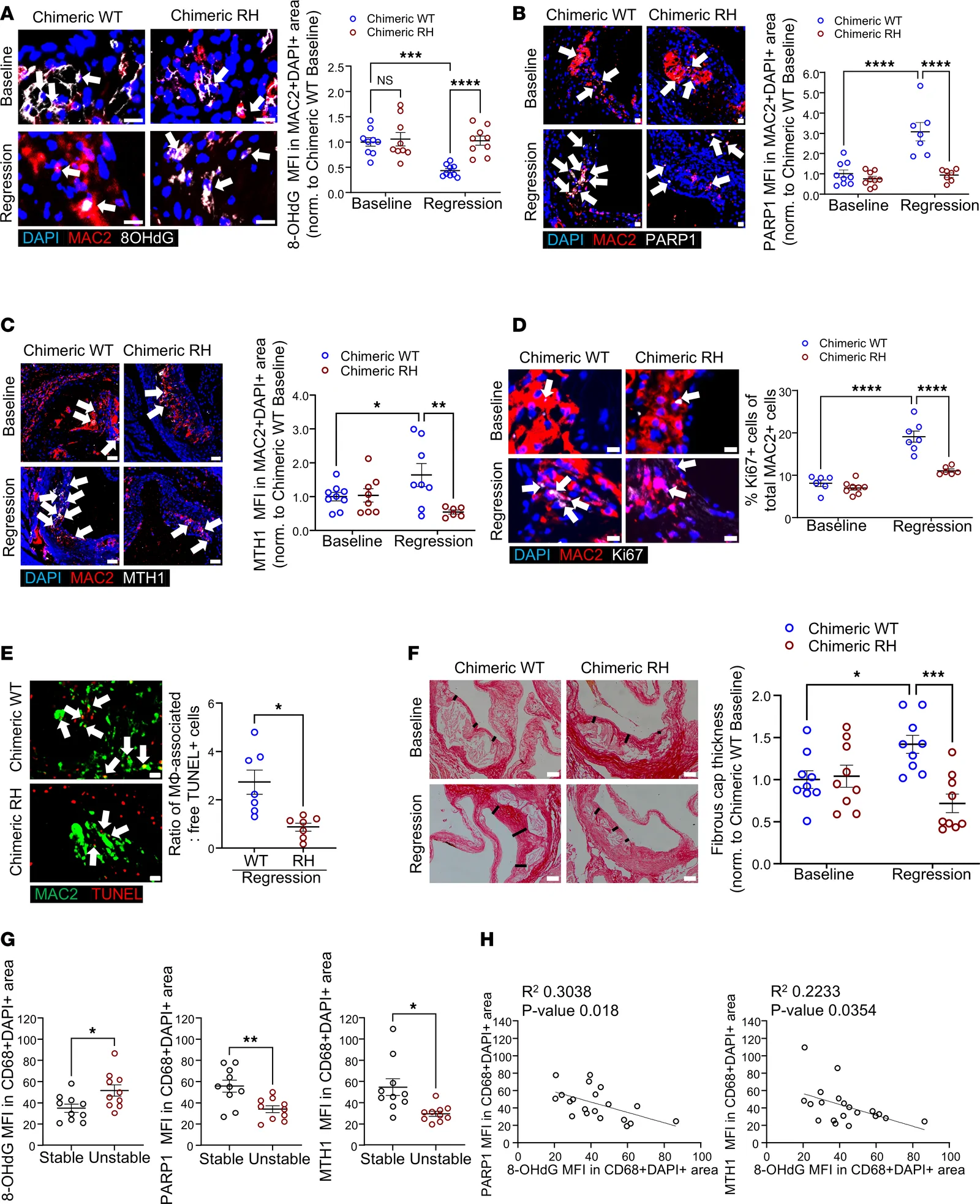
Impaired efferocytosis-induced suppression of oxDNA in atherosclerosis regression in a model of DNMT3A CH. (**A**–**F**) *Ldlr^–/–^* mice were transplanted with a 20%/80% mixture of bone marrow cells from GFP^–^ *Dnmt3a^R878H^* *Csf1rCre^Esr1+^* mice and GFP^+^ WT mice (chimeric RH) or a 20%/80% mixture of bone marrow cells from GFP^–^ and GFP^+^ WT mice (chimeric WT). After 4 weeks, the mice were treated with tamoxifen to induce *Dnmt3a^R878H^* and fed a Western-type diet for 17 weeks. Some mice were harvested (baseline), whereas others were injected with apoB ASO and fed a chow diet for 5 weeks (regression). The aortic root sections were (**A**) immunostained for Mac2 and 8-OHdG (arrows), stained with DAPI, and quantified as the 8-OHdG MFI in Mac2^+^DAPI^+^ areas (*n* = 7–9 mice/group, with similar data in a replicate experiment); (**B**) immunostained for Mac2 and PARP1 (arrows), stained with DAPI, and quantified as the PARP1 MFI in Mac2^+^DAPI^+^ areas (*n* = 7–9 mice/group); (**C**) immunostained for Mac2 and MTH1 (arrows) and stained with DAPI and quantified as the MTH1 MFI in Mac2^+^DAPI^+^ areas (*n* = 7–9 mice/group); (**D**) immunostained for Mac2 and Ki67 (arrows), stained with DAPI, and quantified as the percentage of Ki67^+^ macrophages (*n* = 7–8 mice/group); (**E**) assayed for the ratio of macrophage-associated/free TUNEL^+^ cells (arrows) (*n* = 7 mice/group); and (**F**) stained with Picrosirius red and assayed for fibrous cap thickness (arrows), expressed relative to the WT baseline group (*n* = 9 mice/group). Scale bar: 50 μm. (**G**) Stable and unstable regions of human carotid endarterectomy specimens were immunostained for 8-OHdG, PARP1, and MTH1 and quantified as the 8-OHdG, PARP1, or MTH1 MFI in CD68^+^DAPI^+^ areas (*n* = 10 human specimens). (**H**) Regression analyses of the correlations between macrophage 8-OHdG and PARP1 (left) or MTH1 (right) based on the data in **G**. Scale bars, 50 μm. Data in **A**–**G** indicate the mean ± SEM. *P* values were determined by 2-way ANOVA with Fisher’s LSD post hoc analysis (**A**–**D** and **F**) or 2-tailed Student’s *t* test (**E** and **G**). *P < 0.05, **P < 0.01, ***P < 0.001, and ****P < 0.0001. *R^2^* and *u* values are shown in **H**.

## Version 1 07/21/2026

In-Press Preview

## Version 2 10/15/2026

Electronic publication
