# Supplementary material for: Efferocytosis activates a DNMT3A-mediated oxidized DNA repair pathway to enable tissue resolution
Source: J Clin Invest. 2026 Jul 21;136(20):e203265. doi: 10.1172/JCI203265 (PMC13668614; doi:10.1172/JCI203265)
Supplement: Supplementary file 1 [file jci-136-203265-s029.pdf]

Supplemental Data for

Efferocytosis activates a DNMT3A-mediated oxidized DNA repair pathway to enable tissue resolution

Kleopatra Avrampou[1], Santosh R. Sukka[1], David Ngai[1], Patrick Ampomah[1], Xiaobo Wang[1], George Kuriakose[1], Jacob Glass[2], Bernhard Dorweiler[3], Hanna Winter[4], Lars Maegdefessel[5], Hanrui Zhang[1], Aaron Viny[1], Ira Tabas[1,6,7]

Contents: Supplemental Methods, Supplemental Table, and Supplemental Figures

SUPPLEMENTAL METHODS

Experimental animals. Animal protocols used for experiments were approved by Columbia University's Institutional Animal Care and Use Committee, and animals were cared for according to National Institutes of Health (NIH) guidelines for the care and use of laboratory animals under protocol AABL0571. The mice were housed socially in standard cages at 22°C and under a 12:12 h light/dark cycle in a barrier facility with ad libitum access to water and food. For BMT donor mice for the DEX-thymus, we used 8-week-old *Dnmt3a*$^{fl/fl}$ *Vav1Cre*$^{+/–}$ and *Vav1Cre*$^{+/–}$ control male mice, both on the C57BL/6J background were gifted by Dr. Benjamin Ebert, Dana-Farber Cancer Institute, Harvard Medical School. Eight-week-old male LDLR-KO mice on the C57BL/6J background (B6.129S7-Ldlrtm1Her/J; Jackson Laboratory, strain 002207) were used as BMT-recipient mice for the atherosclerosis experiment, with n numbers indicated in the figure legends. For BMT donor mice for the chimeric atherosclerosis experiment, we used 8-week-old male *Dnmt3a*^{RH}*Csf1rCre*$^{Esr1+}$, or *Csf1rCre*$^{Esr1+}$ and CD45.1 GFP^{+}. *Dnmt3a*^{RH}*Csf1rCre*$^{Esr1+}$ and *Csf1rCre*$^{Esr1+}$ male mice were generated by crossing *Dnmt3a*$^{fl\text{-}R878H}$ (B6(Cg)-Dnmt3atm1Trow/J; Jackson Laboratory, strain 032289) with Tg Csf1r-Mer-iCre-Mer1Jwp or simply *Csf1r*$^{Esr1+}$ (FVB-Tg(Csf1r-cre/Esr1*)1Jwp/J; Jackson Laboratory, strain 019098). CD45.1 GFP^{+} mice were generated by crossing Tg (act-EGFP) Y01Osb mice (C57BL/6-Tg[CAG-EGFP]131Osb/LeySopJ; Jackson Laboratory, strain 006567) with B6 CD45.1 mice (B6.SJL-Ptprca Pepcb/BoyJ; Jackson Laboratory, strain 002014). All purchased mice were allowed to adapt to housing in the animal facility for 1 week before experiments were begun.

Bone marrow transplantation. Recipient mice were irradiated with 10 Gγ with a caesium-137 γ-emitting irradiator (Gamma cell 40, MSD Nordion), and 4-6 hours following irradiation, 2.5 x 10^{6} donor bone marrow cells were injected intravenously by tail vein into each recipient mouse, which were 8-10-week-old C57BL/6J or *Ldlr*$^{-/-}$ male mice (JAX) for the dexamethasone-thymus or atherosclerosis experiments, respectively. The recipient mice were given water containing 10 mg ml^{-1} neomycin and allowed to recover for 4 weeks before initiating the experiments.

Dexamethasone-thymus assay. Mice were injected intraperitoneally with 250 μg dexamethasone (DEX; Sigma) in PBS or an equal volume of PBS (control), and 4 or 18 hours after injection, the mice were sacrificed. One lobe of the thymus was mechanically dissociated, and the cells were counted to determine the total cell number. The other lobe of the thymus was harvested and fixed in 10% formalin, embedded in paraffin, sectioned at a thickness of 5 μm, and analyzed by H&E or immunofluorescence staining. Necrotic area was quantified by using H&E-

stained sections and measuring the percentage area of regions that are hypocellular or contain fragmented nuclei. In situ efferocytosis was quantified as the ratio of Mac2$^+$ (macrophage)-associated TUNEL$^+$ cells to free TUNEL$^+$ cells. To determine whether PARP1 and MTH1 were required for efferocytosis-induced suppression of oxDNA in this model, C57BL/6J mice were administered the PARP1 inhibitor PJ34 (25 mg/kg; HY-13688A), the MTH1 inhibitor TH287 (5 mg/kg; HY-16965), or DMSO vehicle control. Compounds were delivered intraperitoneally 30 minutes before and both 15 minutes and 2 hours after DEX injection. Thymi were collected 4 hours after DEX administration. To assess the role of MTH1 in thymic repair and EIMP, the thymic of mice subjected to the TH287 treatment protocol were harvested 18 hours after DEX and assayed for these two endpoints.

Generating mouse bone marrow-derived macrophages (BMDMs). Bone marrow cells were isolated from the femurs of 8–12-week-old male C57BL/6J mice, *Dnmt3a*$^{fl/fl}$*Vav1Cre*$^{+/–}$, and Vav1Cre$^{+/–}$ mice. Bone marrow cells were cultured for 7 days in BMDM differentiation medium, which is composed of DMEM supplemented with 10% (v/v) heat-inactivated FBS, 100 U mL^{-1} penicillin-streptomycin, and 20% (v/v) L-929 cell-conditioned medium. Cells were incubated at 37°C in a 5% CO2 incubator.

Generating human monocyte-derived macrophages (HMDMs). Peripheral blood mononuclear cells were isolated from the buffy coats of anonymous healthy adult donors with informed consent (New York Blood Center) using Histopaque-1077 (Sigma) cell separation medium. The isolated cells were rinsed and cultured for 7-14 days in RPMI-1640 medium with L-glutamine (10-040; Corning) supplemented with 10% (v/v) heat-inactivated FBS, 100 U mL^{-1} penicillin-streptomycin, and 10 ng/mL human M-CSF (Peprotech). Experiments were conducted in naive M-CSF macrophages. The cells were cultured in a humidified CO2 incubator at 37°C.

Cell lines. L-929 fibroblasts (CCL-1), Jurkat T-lymphocytes (ATCC #TIB-152), 293T epithelial cells (ATCC #CRL-3216) were purchased from ATCC. All cell lines were cultured in DMEM supplemented with 10% heat-inactivated fetal bovine serum (FBS) and 100 U mL^{-1} penicillin-streptomycin (Gibco). The cells were cultured in a humidified CO2 incubator at 37°C.

Generating apoptotic cells (ACs) and incubating with BMDMs. Jurkat cells were resuspended in PBS and irradiated with a 254-nm UV lamp for 15 min to induce apoptosis. To fluorescently label these ACs, the irradiated ACs were pelleted by centrifugation, resuspended in PBS, mixed with Diluent C containing PKH26 (red) (Sigma), or CellVue Claret (far red) (Sigma), and incubated at 37°C for 5 min. The labelling reaction was quenched by adding an equal volume of heat-inactivated FBS. ACs were pelleted, resuspended in PBS, and incubated at 37°C for 2-3 hours.

To generate apoptotic HMDMs, differentiated HMDMs were treated with 1 μM staurosporine (Sigma) in RPMI-1640 supplemented with 10% (v/v) heat-inactivated FBS and 100 U mL-1 penicillin-streptomycin for 48 hours. Apoptotic HMDMs were then collected in Cellstripper (Corning) with gentle scraping. The ACs were then pelleted, resuspended in fresh PBS, and added to macrophages at a 5:1 number ratio of ACs:macrophages and incubated for up to 45 min at 37°C. Unbound ACs were then washed off with PBS and chased for 1 or 24 hours in DMEM supplemented with 10% (v/v) -FBS, 100 U mL^{-1} penicillin-streptomycin, and 20% (v/v) L-929 cell-conditioned medium with or without the addition of the specified inhibitors.

siRNA transfection. Macrophages were seeded at a confluence of ~70% confluency in BMDM differentiation medium. The cells were transfected with 50 nM scrambled siRNA or targeted siRNA (Dharmacon ON-TARGETplus SMARTpool siRNA or IDT DsiRNAs) in Opti-MEM reduced serum medium (Gibco) with Lipofectamine RNAiMAX (Life Technologies). After 18-20 hours, the medium was changed to fresh BMDM differentiation medium and incubated for an additional 54 hours before use.

Cell counting. Macrophages were seeded at 100,000 cells/well in a 12-well plate in full medium and then incubated with or without ACs for 45 min and chased for 24 hours. The macrophages were then detached with 10 mM EDTA in ice-cold PBS by gentle scraping. Collected cells were counted using a Countess II Automated Cell Counter (Invitrogen).

Immunoblotting. For preparation of chromatin-bound proteins and soluble nuclear extracts, BMDMs were washed in ice-cold PBS, and 1×10^6 cells were processed using the Subcellular Protein Fractionation Kit for cultured cells (Pierce 78840) according to the manufacturer's instructions. After quantifying protein content using the Pierce BCA (bicinchoninic acid) assay, the lysates were diluted in 2x or 4x Laemmli sample buffer (Bio-Rad) with 50 mM β-mercaptoethanol (Sigma) and boiled at 95-100°C for 5 min, loaded onto 4-20% SDS-PAGE gradient gels (Invitrogen), and run at 120V for 90 min. Protein was then transferred onto a 0.45-micron nitrocellulose membrane at 300 mA for 90 min or at 90 mA overnight. The membranes were then blocked for 1 hour with 5% skim milk or 5% bovine serum albumin in Tris-buffered saline with Tween-20 (TBST), followed by incubation overnight at 4°C with primary antibody diluted in 5% skim milk or 5% bovine serum albumin. The membranes were then washed 3 x 5 min with TBST, incubated with HRP-conjugated secondary antibodies at room temperature for 1-2 hours, and washed 3 x 5 min with TBST. The following antibodies were used: rabbit anti-PARP1 (CST 9532; 1:1,000), rabbit anti- MTH1 (Novus NB100-109SS; 1:1,000), rabbit anti-DNMT3A (CST3598; 1:1,000), anti-rabbit IgG HRP-linked (CST 7074; 1:2,000).

Immunofluorescence staining and microscopy. BMDMs or HMDMs were seeded on 8-well chamber slides at 30,000 cells per well. The next day, PKH26-labelled ACs were added to the cell monolayers for 45 min and then chased for 1, 3, 6 or 24 hours before conducting the indicated endpoint assays. In some experiments, the macrophages were pretreated with 20 mM PJ34 (HY-13688A), 20 mM TH287 (HY-16965), or 20 mM Aza-C (HY-10586) for 2 hours before adding the ACs. For the EdU incorporation assay, cells were pretreated with 20 μM EdU (iFluor 488, ab219801) for 2 hours. The cells were fixed with 4% PFA for 10 min at room temperature, washed with PBS, permeabilized with 0.3% Triton-X (Sigma), and blocked with 5% bovine serum albumin for 1 hour at room temperature. The fixed cells were then incubated with goat anti-8-OHdG (Millipore Sigma AB 5830; 1:200), mouse anti-PARP1 (Proteintech 66520;1:200), rabbit anti-MTH1 (Proteintech 16705;1:100), rabbit anti-5-methyl C (CST 28692; 1:200), rabbit anti-p21 (CST 39256;1:100), mouse anti-p53 (CST 2324;1:100), rabbit anti-γH2AX (CST 5438;1:100), rabbit anti-OGG1 (Proteintech 15125-1-AP;1:100) diluted in blocking solution overnight at 4°C. On the following day, the cells were washed 3 x 5 min with PBS, incubated with fluorescently labeled secondary antibodies for 1 hour, washed, and counterstained with Hoechst (CST). Images were taken using a NIKON A1 confocal microscope to quantify nuclear expression or Leica epifluorescence microscope (DMI6000B) to analyze total expression. Image analysis was conducted using ImageJ quantification software.

For paraffin-embedded tissues, sections were deparaffinized with xylene, rehydrated with 100% ethanol and 70% ethanol, and rinsed with PBS. Antigen retrieval was conducted using 1x citrate-based antigen unmasking solution (Vector 330) or 1x antigen retrieval tris-EDTA buffer pH 9.0 (Abcam AB93684) together with pressure cooking for 10 min. TUNEL staining was conducted after antigen retrieval using the In Situ Cell Death Detection Kit (TRM Red Sigma) according to the manufacturer's instructions. Tissue sections were then blocked with 2% BSA in PBS for 1 hour at room temperature, followed by incubation with primary antibodies diluted in blocking solution overnight at 4°C. The following primary antibodies were used: rat anti-Mac2 (Cedarlane CL8942LE; 1:500), goat anti-8-OHdG (Millipore Sigma AB 5830; 1:200), mouse anti-PARP1 (Proteintech 66520;1:100), rabbit anti-MTH1 (Proteintech 16705;1:100), rabbit anti-arginase 1 (Proteintech 16001;1:100), rabbit anti-CD3 (Proteintech 17617; 1:100), rabbit anti-TGF-β1 (Abcam EPR21143; 1:50), rabbit anti-FoxP3 (Novus NB100-39002;1:100), and rabbit anti-lumican (Abcam EPR8898;1:100). Tissues sections were washed 3 x 5 min with PBS and then incubated for 1 hour at room temperature in the dark with fluorescently labelled secondary antibodies diluted in blocking solution. Tissues were imaged using a Leica epifluorescence (DM16000B) or NIKON A1 microscope. The following secondary antibodies were used: donkey

anti-goat AF488 (Invitrogen A110055; 1:200), donkey anti-rabbit AF555 (Invitrogen A31572; 1:200), donkey anti-rabbit AF488 (Invitrogen A21206; 1:200), donkey anti-rat AF647 (Invitrogen A21247, 1:200), donkey anti-mouse AF594 (Invitrogen A21203), donkey anti-mouse AF488 (Invitrogen A21202), donkey anti-sheep AF488 (Invitrogen A11015), donkey anti-sheep AF594 (Invitrogen A11015).

Reverse transcription-quantitative polymerase chain reaction (RT-qPCR). RNA was isolated from macrophages using the PureLink RNA Mini kit (Life Technologies) according to the manufacturer's protocol. The RNA quality and concentration were measured using a NanoDrop spectrophotometer (ThermoFisher). cDNA synthesis was generated from RNA using oligo(dT) and Superscript II (Applied Biosystems). RT-qPCR was conducted using the QuantStudio 3 Real-Time PCR system (Applied Biosystems) and the Power SYBR Green PCR Master Mix (Applied Biosystems). The primers are listed in Supplementary Table 1. Expression of genes of interest was normalized to the expression of the housekeeping gene *Hprt*.

Atherosclerosis experiments and plaque analysis. $Ldlr^{-/-}$ mice were transplanted with bone marrow cells from $Dnmt3a^{RH}Csf1rCre^{Esr1+}$ (GFP^{-}) mice or control $Csf1rCre^{Esr1+}$ (GFP^{+}) mice at a 20:80 ratio. After 4 weeks, each mouse was injected 3 times subcutaneously over 1 week with 5 mg tamoxifen (Sigma) dissolved in 200 ml of warm corn oil and then fed a Western-type diet (Envigo, TD88137) for 16 weeks. One set of mice of each genotype was harvested at this point (baseline), while another set of mice from each genotype was injected intraperitoneally with apolipoprotein B-antisense oligonucleotide (ApoB ASO) and switched to chow diet for an additional 5 weeks. Apo-B ASO was provided from Ionis (#147764), and mice were injected at 100 mg/kg per week for a total of 5 weeks. Before harvesting, each group of mice was fasted overnight, and blood glucose was assayed using the OneTouch Ultra device. After sacrifice, blood was collected by cardiac puncture and used for complete blood counts, plasma cholesterol measurements, and flow cytometry analysis for GFP. Blood cell counts were measured by the FORCYTE Hematology Analyzer (Oxford Science) or VetScan HM5, and the plasma cholesterol was measured using a Wako Diagnostics kit. Aortic roots were histologically processed for plaque analysis. Paraffin-embedded sections of the aortic roots were stained for H&E to quantify the non-cap lesion and necrotic core areas and with picrosirius red (Polysciences; 24901A) to quantify fibrous cap thickness. The remaining sections were used for immunofluorescence analysis.

Human atherosclerotic plaque analysis. De-identified atherosclerotic plaques from elective endarterectomy procedures were obtained with informed consent and then immediately snap-frozen in the operating room using liquid nitrogen. Gross dissection of specimens into unstable

and stable regions was done based on macroscopic appearance. Unstable regions were identified as dark brown, irregular areas, whereas stable regions were smoother and lighter in color. We showed previously that unstable regions defined by these criteria correlated with thinner fibrous caps, larger areas of plaque necrosis, higher oxidative stress, and higher ratio of pro-inflammatory:pro-resolving lipid mediators (1). To analyze human plaque histology, unstable and stable plaques from each donor were fixed using 10% formalin overnight at room temperature (RT) before decalcification, paraffin embedding, and sectioning. The distinction between unstable and stable regions of the plaques were first validated by picrosirius red staining, i.e., to verify thinner fibrous caps in the unstable regions (1), and then immunostained for macrophages (CD68), 8-OHdG, PARP1, and MTH1.

Cloning and retrovirus production. pMYs-IRES-GFP retroviral vector was obtained from Cell Biolabs, Inc. (#RTV-021), pcDNA3-Tet1 from Addgene (#60938), and Myc-DDK-tagged-Nudt1 from Origene (#MR201165). Tet1 and Nudt1 ORF fragments were prepared from pcDNA3-Tet1 and Nudt1 (Myc-DDK-tagged), respectively, and inserted into the retroviral vector pMY. pMY vectors containing Tet1 or Nudt1 were transfected into 293T cells (#CRL-3216, ATCC). The collected supernatants were filtered using a 0.45 μm-filter Millipore® Steriflip® conical tube, mixed with Retro-X concentrator (Takara #631455) in a ratio of 1 Retro-X concentrator:3 viral supernatant, and incubated overnight at 4^0C. The mixtures were then centrifuged at 2000xg for 45 min at 4^0C, and the viral pellets were collected.

Retrovirus transduction of hematopoietic stem cells and generation of macrophages. RetroNectin®-coated plates (20 μg/ml; Takara #T100A/B) were prepared according to the manufacturer's instructions. The concentrated virus supernatant fractions were added into the coated plates, which were then centrifuged at 2000xg for 90 min at 32^0C. Hematopoietic stem cells were isolated from 7- or 8-week-old male mice using the EasySep™ mouse hematopoietic progenitor cell isolation kit (#19856) and were plated on the RetroNectin®-coated plates after the virus-binding step. The cells were cultured for 7 days in macrophage differentiation medium, which is composed of DMEM supplemented with 20% (v/v) heat-inactivated FBS, 100 U mL^{-1} penicillin-streptomycin, 1x GlutaMAX 100x (Gibco # 35050061), 1x non-essential amino acids (NEAA) 100x MEM Non-Essential Amino Acids Solution (Gibco # 11140050), 55 nM 2-mercaptoethanol (ThermoFisher #125470025), and 10 ng/mL recombinant M-CSF (mouse Peprotech 3125-02).

Flow cytometry analysis. To obtain single-cell suspensions of white blood cells, peripheral blood was collected from each mouse, and red blood lysis was performed using ACK lysis buffer (Quality Biological 118-156-101). After two washes with 1x PBS (Gibco) supplemented with 4%

heat-inactivated FBS, the cells were passed through a 40-μm filter and sorted using a Fortessa cell sorter (BD Biosciences). Analysis was conducted using FlowJo software; dead cells were excluded by DAPI staining. Cellular reactive oxygen species (ROS) were measured by CellROX™ (Life Technologies). In brief, cells were stained with 500 nM CellROX™ reagent, detached with Cellstripper (Corning) with gentle scraping, and resuspended in 4% PFA for 10 min in the dark. The cells were then rinsed with 1x PBS wash and immediately analyzed by flow cytometry using a Fortessa cell sorter and FlowJo software.

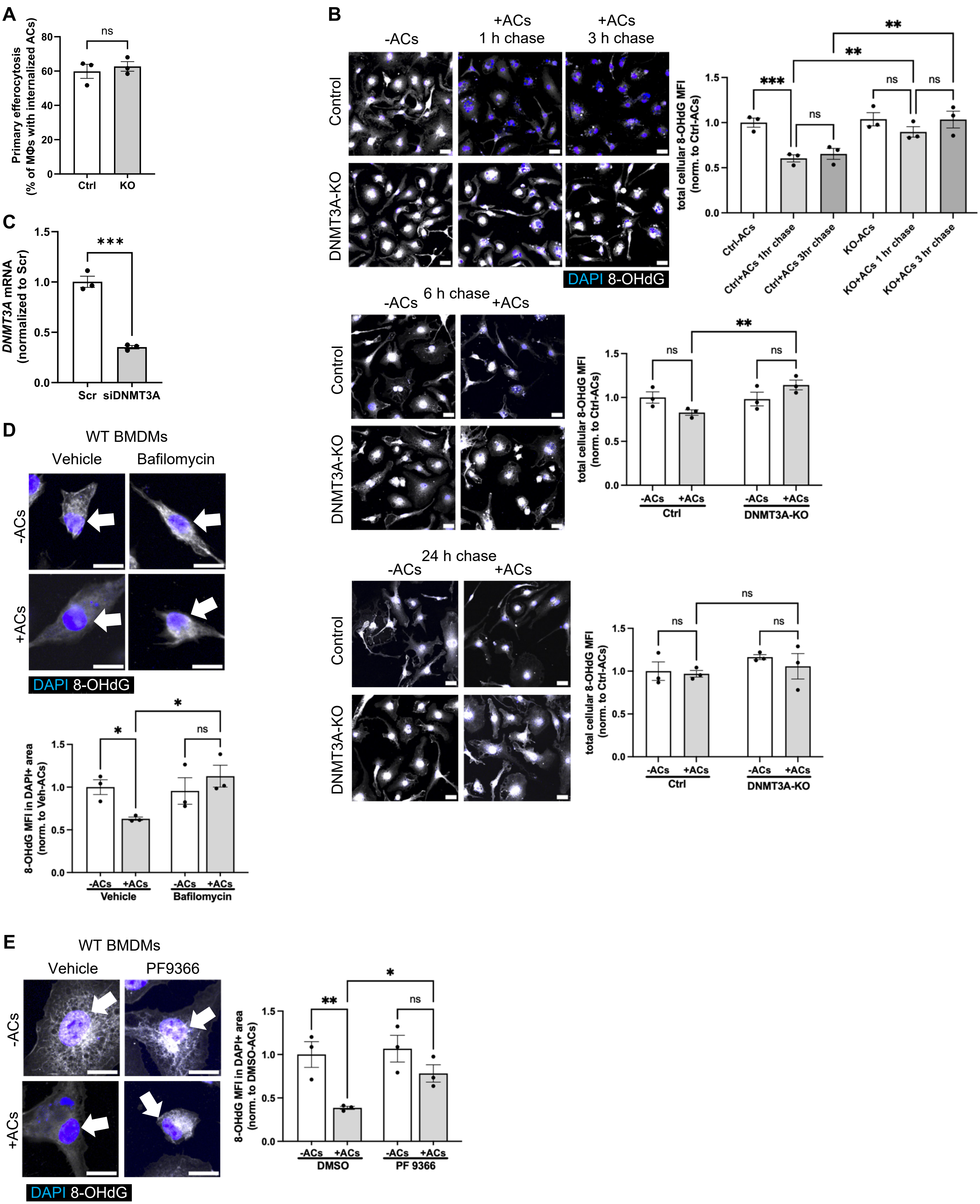
A
Primary efferocytosis
(% of MΦs with internalized ACs)
ns
Ctrl
KO
B
-ACs
+ACs
1 h chase
+ACs
3 h chase
Control
DNMT3A-KO
DAPI 8-OHdG
total cellular 8-OHdG MFI
(norm. to Ctrl-ACs)
***
**
**
ns
Ctrl-ACs
Ctrl+ACs 1hr chase
Ctrl+ACs 3hr chase
KO-ACs
KO+ACs 1 hr chase
KO+ACs 3 hr chase
6 h chase
-ACs
+ACs
Ctrl
DNMT3A-KO
24 h chase
C
DNMT3A mRNA
(normalized to Scr)
***
Scr
siDNMT3A
D
WT BMDMs
Vehicle
Bafilomycin
8-OHdG MFI in DAPI+ area
(norm. to Veh-ACs)
*
E
PF9366
8-OHdG MFI in DAPI+ area
(norm. to DMSO-ACs)
DMSO
PF 9366

Supplemental Figure 1. Supplemental data related to Efferocytosis induces suppression of oxidized DNA in a DNMT3A-dependent manner. (**A**) BMDMs were incubated with PKH26-labeled ACs for 45 min and then quantified by fluorescence microscopy for the percentage of total macrophages that were PKH26$^+$ as a measure of primary efferocytosis (*n* = 3 replicates/group). (**B**) BMDMs from WT (Control) or DNMT3a-KO mice were incubated without (-ACs) or with apoptotic cells (+ACs) for 45 minutes, after which non-internalized ACs were removed by rinsing. After an additional 1, 3, 6, and 24 hours of incubation, the cells were immunostained for 8-OHdG, and total cellular levels of 8-OHdG were quantified for 8-OHdG MFI of each macrophage (n = 3 replicates/group). Scale bar, 50 μm. (**C**) HMDMs were transfected with scrambled RNA (Scr) or siDNMT3A, and *DNMT3A* mRNA was measured by RT-qPCR (*n* = 3 replicates/group). **(D**) As in Figure 1E, but WT BMDMs were pre-treated for 2 hours with vehicle (DMSO) or 500 nM bafilomycin-A1. Arrows indicate macrophage nuclei. The cells were then quantified for 8-OHdG MFI in DAPI+ areas (n = 3 replicates/group). Scale bar, 50 μm. (**E**) As in (D), except the macrophages were pre-treated for 2 hours with vehicle (DMSO) or 10 μM of the MAT2A inhibitor PF9366. The cells were then quantified for 8-OHdG MFI in DAPI+ areas (n = 3 replicates/group). Arrows indicate macrophage nuclei. Scale bar, 50 μm. Data are shown as mean ± s.e.m., and *P* values were determined by the Student's t-test for the panels A and C, one-way for panel B (1h and 3h) or two-way ANOVA for panel A (6h and 24h) and E, with Fisher's LSD post-hoc analysis *P < 0.05; **P < 0.01; ***P < 0.001; ****P<0.0001; ns, non-significant.

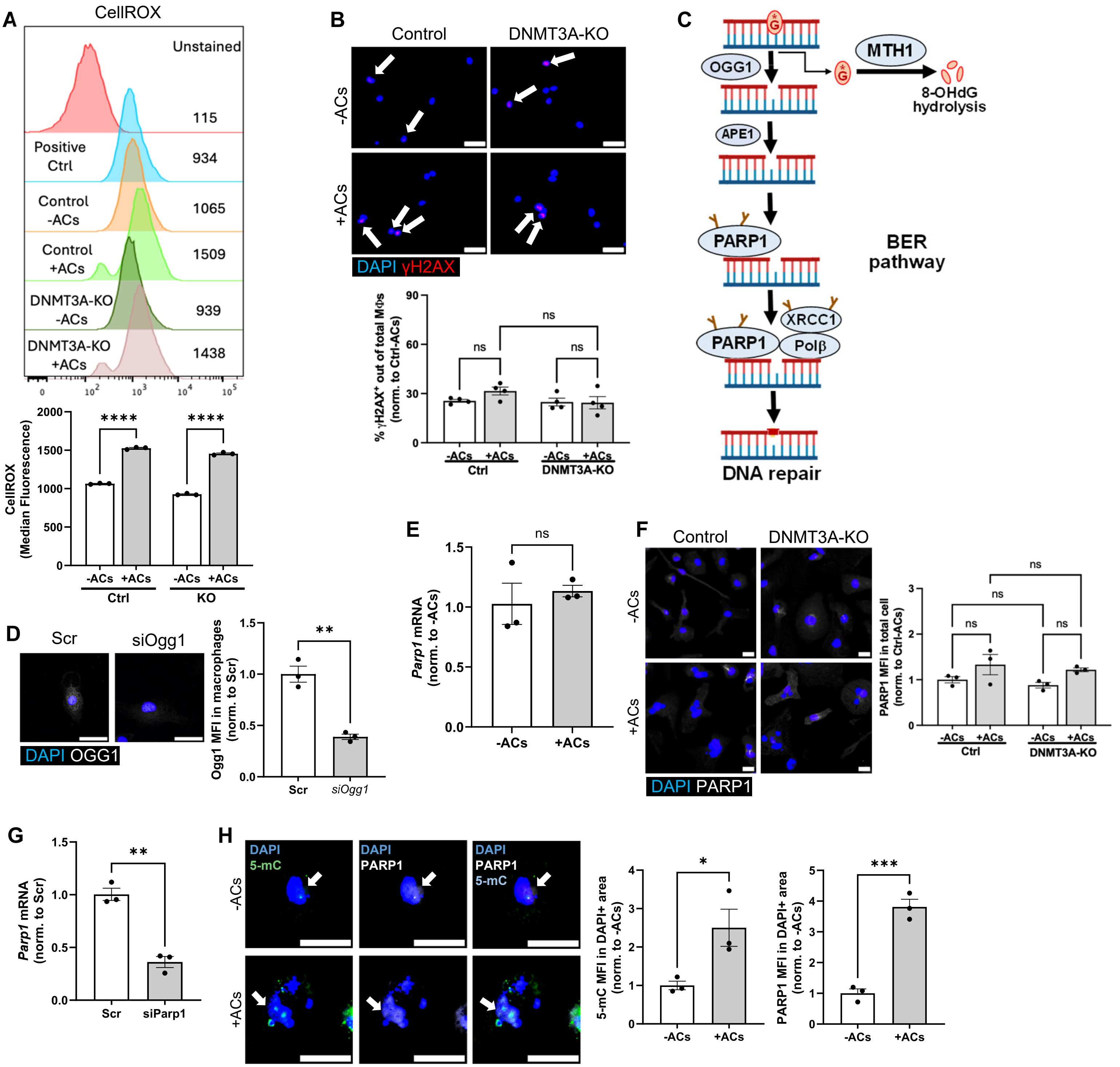

A
CellROX
Unstained 115
Positive Ctrl 934
Control -ACs 1065
Control +ACs 1509
DNMT3A-KO -ACs 939
DNMT3A-KO +ACs 1438
CellROX (Median Fluorescence)
-ACs +ACs -ACs +ACs
Ctrl KO
B
Control DNMT3A-KO
-ACs
+ACs
DAPI γH2AX
% γH2AX+ out of total MΦs (norm. to Ctrl-ACs)
Ctrl DNMT3A-KO
C
OGG1
MTH1
8-OHdG hydrolysis
APE1
PARP1
BER pathway
XRCC1
Polβ
DNA repair
D
Scr siOgg1
DAPI OGG1
Ogg1 MFI in macrophages (norm. to Scr)
E
Parp1 mRNA (norm. to -ACs)
F
Control DNMT3A-KO
DAPI PARP1
PARP1 MFI in total cell (norm. to Ctrl-ACs)
G
Parp1 mRNA (norm. to Scr)
Scr siParp1
H
DAPI 5-mC
DAPI PARP1
DAPI PARP1 5-mC
5-mC MFI in DAPI+ area (norm. to -ACs)
PARP1 MFI in DAPI+ area (norm. to -ACs)

Supplemental Figure 2. Supplemental data related to the repair of oxDNA in efferocytosing macrophages. (**A**) BMDMs from WT (Control) or DNMT3A-KO mice were incubated without ACs (-ACs) or with ACs (+AC) for 45 min, after which non-internalized ACs were removed by rinsing. After an additional 1-hour incubation, the macrophages were assayed for CellROX™ fluorescence by flow cytometry (unstained; positive control using tert-butyl hydroperoxide) (*n* = 3 replicates/group). (**B**) As in (A), but the cells were immunostained for γH2AX, and the percentage of γH2AX+ macrophages was quantified using immunofluorescence microscopy (n = 4 replicates/group). Arrows indicate $\gamma H2AX^+$ macrophages. Scale bar, 50 μm. (**C**) Schematic representation of base-excision repair (BER) pathway for oxDNA. (**D**) As in Figure 2A, but the macrophages were immunostained for OGG1, and quantified for OGG1 MFI in MΦ (*n* = 3 replicates/group). Scale bar 50 μm. (**E**) BMDMs from WT mice were incubated without ACs (-ACs) or with ACs (+AC) for 45 min, after which non-internalized ACs were removed by rinsing. After an additional 1-hour incubation, the macrophages were assayed for *Parp1* mRNA by RT-qPCR (*n* = 3 replicates/group). (**F**) As in (A), but macrophages were immunostained for PARP1 and quantified for PARP1 MFI in total cell (*n* = 3 replicates/group). Scale bar 50 μm. (**G**) WT BMDMs were transfected with scrambled RNA or siParp1, and *Parp1* mRNA was quantified by RT-qPCR (*n* = 3 replicates/group). (**H**) As in (E), but the macrophages were assayed by confocal immunofluorescence microscopy for 5-methylcytosine (5-mC) and nuclear PARP1 (*n* = 3 replicates/group). Scale bar, 50 μm. Data are shown as mean ± s.e.m., and *P* values were determined by the Student's t-test for panels D, E, G, and H or two-way ANOVA for panels A, B, and F, with Fisher's LSD post-hoc analysis. *P < 0.05; **P < 0.01; ***P < 0.001; ****P < 0.0001; ns, non-significant.

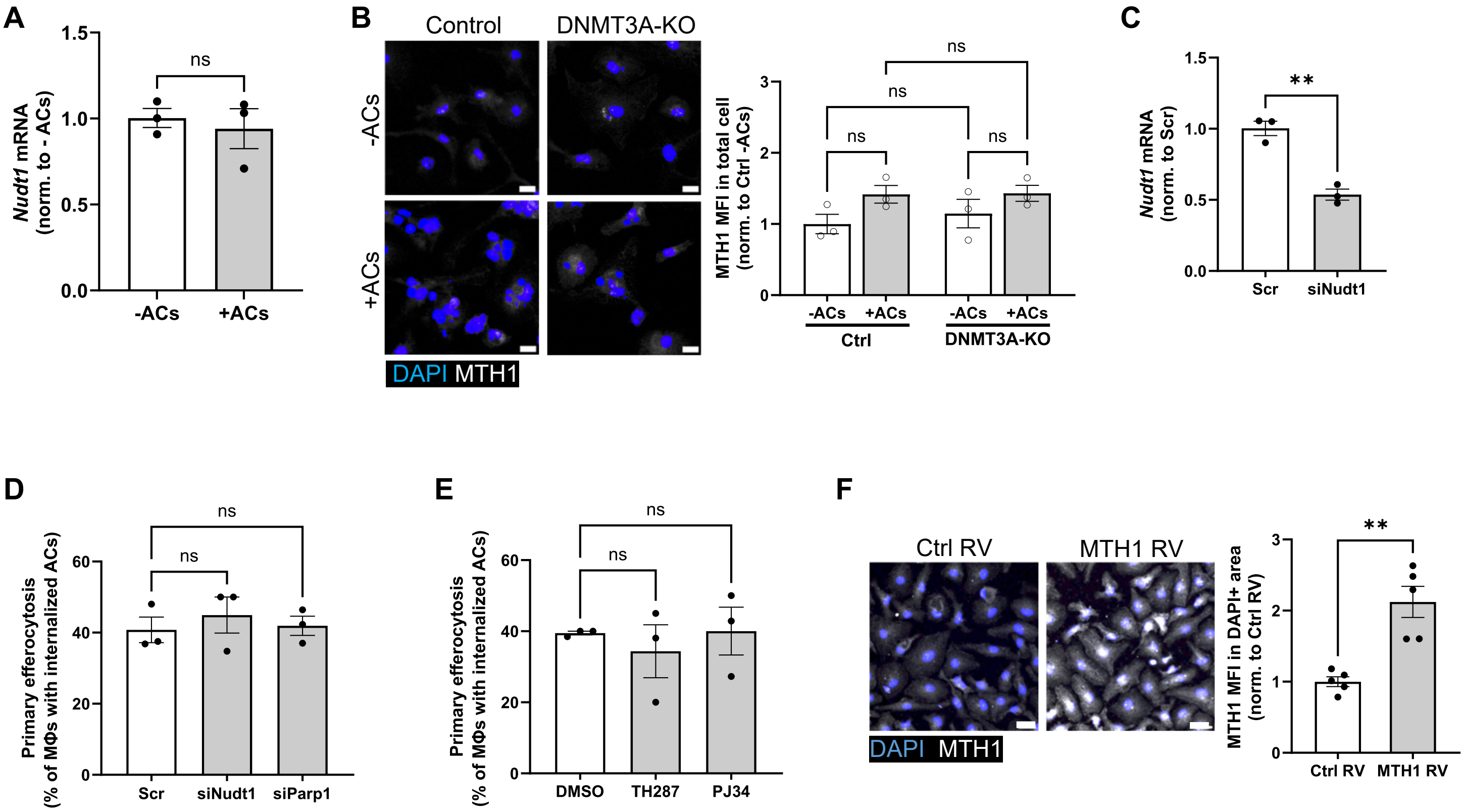

Supplemental Figure 3. Supplemental data related to PARP1/MTH1-mediated repair of oxDNA in efferocytosing macrophages. (**A**) BMDMs from WT mice were incubated without ACs (-ACs) or with ACs (+AC) for 45 min, after which non-internalized ACs were removed by rinsing. After an additional 1-hour incubation, the macrophages were assayed for *Nudt1* mRNA (gene name for MTH1) by RT-qPCR (*n* = 3 replicates/group). (**B**) The same macrophages in Supplemental Figure 2F were immunostained for MTH1 and quantified for MTH1 MFI in total cell (*n* = 3 replicates/group). Scale bar 50 μm. (**C**) WT BMDMs were transfected with scrambled RNA or siNudt1, and *Nudt1* mRNA was quantified by RT-qPCR (*n* = 3 replicates/group). (**D**) WT BMDMs transfected with scrambled RNA, siParp1, or siNudt1 were incubated with PKH26-labeled ACs for 45 min and then quantified by fluorescence microscopy for the percentage of total macrophages that were PKH26$^+$ as a measure of primary efferocytosis (*n* = 3 replicates/group). (**E**) WT BMDMs pretreated for 2 hours with 20 μM PJ34, 20 μM TH287, or vehicle control (DMSO) were incubated with PKH26-labeled ACs for 45 min and then quantified by fluorescence microscopy for the percentage of total macrophages that were PKH26$^+$ as a measure of primary efferocytosis (*n* = 3 replicates/group). (**F**) The control (Ctrl RV) and MTH1-transduced (MTH1 RV) macrophages from Figure 3H were immunostained for MTH1, stained for DAPI, and quantified for MTH1 in DAPI$^+$ areas (*n* = 3 replicates/group). Scale bar, 50 μm. Data are shown as mean ± s.e.m., and *P* values were determined by the Student's t-test for panels A, C, and F, two-way ANOVA for panel B, or one-way ANOVA for panels D and E, with Fisher's LSD post-hoc analysis. **P < 0.01; ns, non-significant.

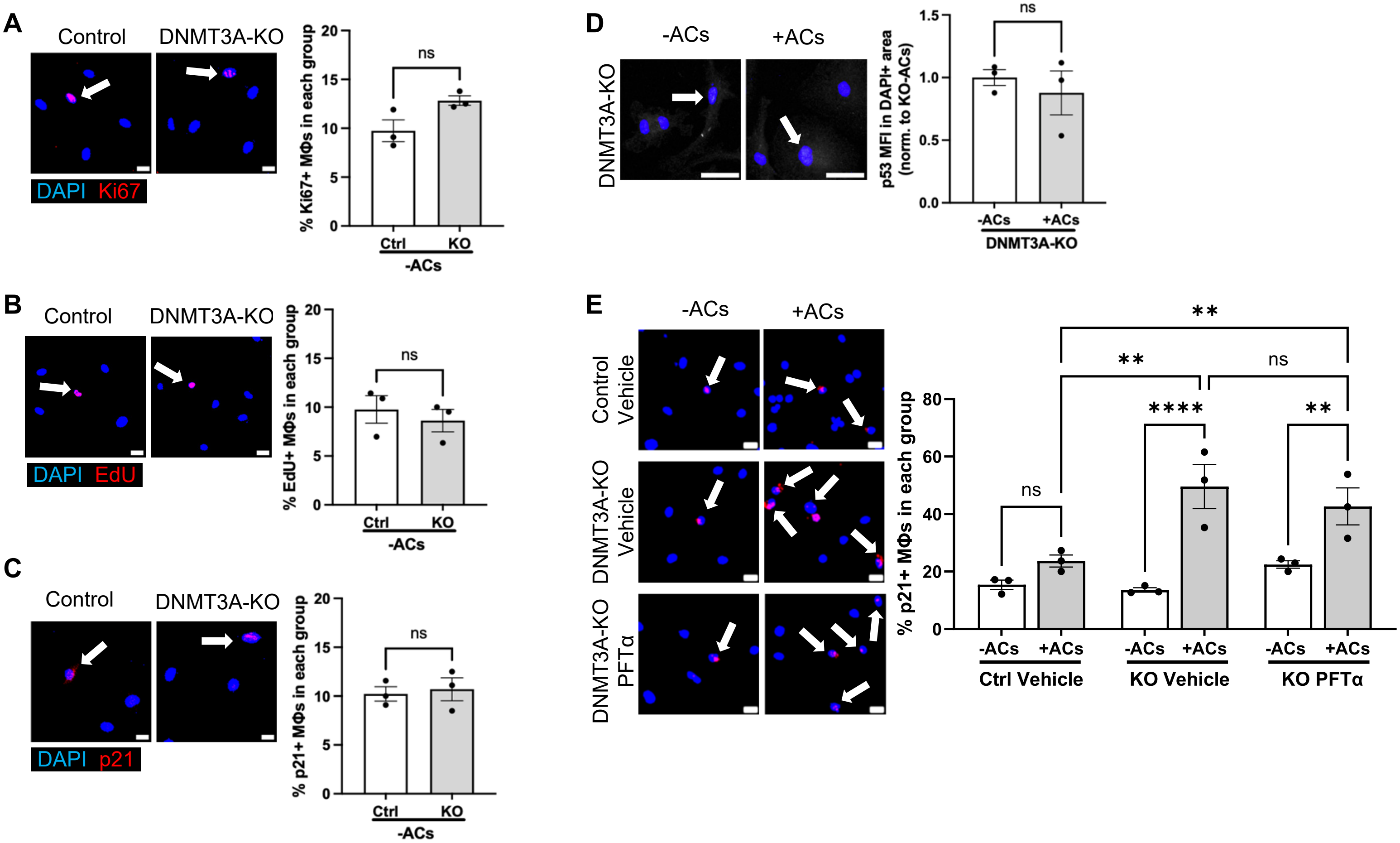

Supplemental Figure 4. Supplemental data related to the role of efferocytosis-induced suppression of oxDNA in macrophage proliferation. (**A**) Similar to Figure 4A, but the percentage of Ki67+ macrophages in –AC groups was quantified using immunofluorescence microscopy (n = 3 replicates/group). Arrows indicate macrophage nuclei positive for Ki67. Scale bar, 50 µm. (**B**) Similar to Figure 4B, but the percentage of EdU+ macrophages in –AC groups was quantified using immunofluorescence microscopy (n = 3 replicates/group). Arrows indicate macrophage nuclei positive for EdU. Scale bar, 50 µm. (**C**) Similar to Figure 4C, but the percentage of p21+ macrophages in –AC groups was quantified using immunofluorescence microscopy (n = 3 replicates/group). Arrows indicate macrophage nuclei positive for p21. Scale bar, 50 µm. (**D**) BMDMs from DNMT3A-KO mice were incubated without ACs (-ACs) or with ACs (+AC) for 45 min, after which non-internalized ACs were removed by rinsing. After an additional 21-hour incubation, macrophages were immunostained for p53 and quantified for p53 in DAPI$^+$ areas (*n* = 3 replicates/group). Arrows indicate macrophage nuclei. Scale bar 50 µm. (**E**) Similar to (D), but the macrophages were treated with 10 µM PFTα for additional 24 hours of incubation, and the percentage of p21+ macrophages in –AC and +AC groups was quantified using immunofluorescence microscopy (n = 3 replicates/group). Data are shown as mean ± s.e.m., and *P* values were determined by the Student's t-test for panels A-D or one-way ANOVA for panel E, with Fisher's LSD post-hoc analysis. **P < 0.01; ****P < 0.0001; ns, non-significant.

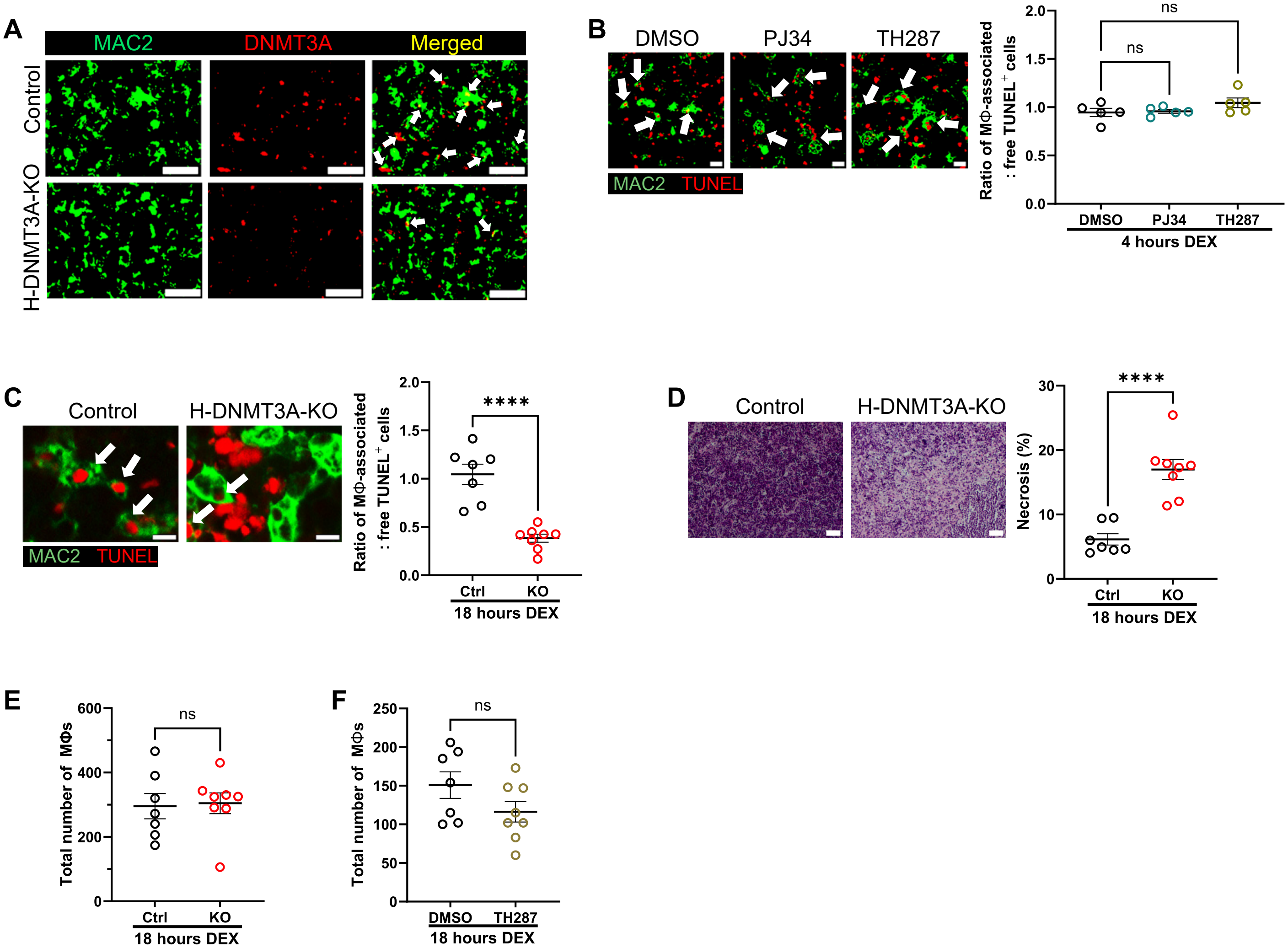

Supplemental Figure 5. Supplemental data related to the role of the DNMT3A-mediated oxDNA repair pathway in the DEX-thymus model. (**A**) Representative images of Ctrl and KO thymi were immunostained for Mac2 (macrophages, green) and DNMT3A (red). The arrows in the merged images show macrophages expressing DNMT3A. Scale bar, 50 μm. (**B**) The thymi of the PJ34-treated, TH287-treated, and control mice from Figure 5F were quantified for the ratio of macrophage-associated:free TUNEL$^+$ cells as a measure of primary efferocytosis (arrows) (*n* = 5 mice/group). Scale bar, 50 μm. (**C-E**) The 18-hour post-DEX thymi from the control and DNMT3A-KO mice in Figure 5G were quantified for (**C**) the ratio of macrophage-associated:free TUNEL$^+$ cells (arrows) as a measure of continuing efferocytosis (Scale bar, 50 μm); (**D**) the percent necrotic area in H&E-stained sections (Scale bar, 50 μm); and (**E**) the total number of macrophages per section (*n* = 7-8 mice/group). (**F**) The thymi of the control and TH287-treated mice in Figure 5I were assayed for the total number of macrophages per section (*n* = 7-8 mice/group). Data are shown as mean ± s.e.m., and *P* values were determined by one-way ANOVA with Fisher's LSD post-hoc analysis for panel B or the Student's t-test for panels C-F. ****$P < 0.0001$; ns, non-significant.

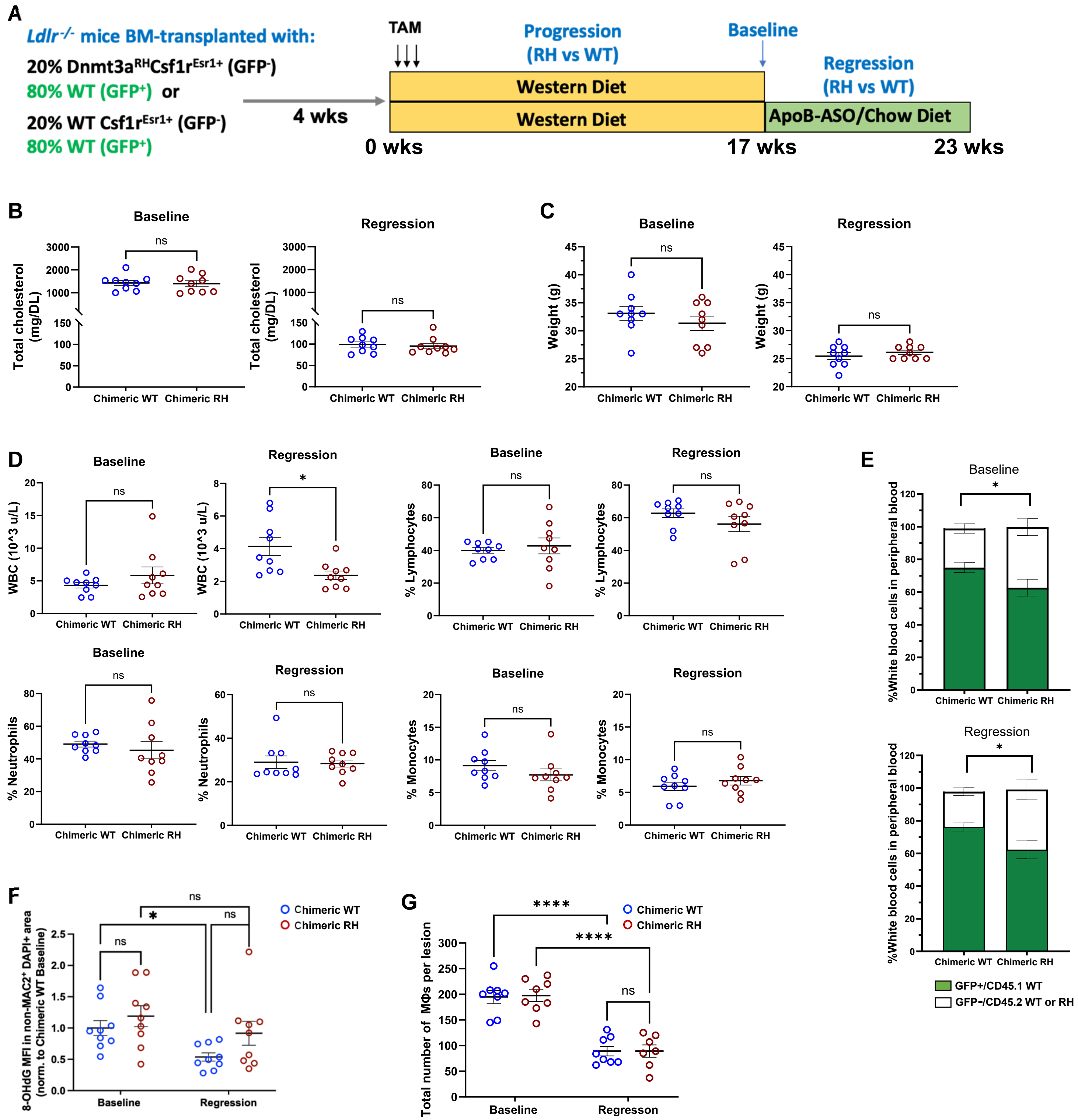

Supplemental Figure 6. Supplemental data related to the effect of DNMT3A-CH on oxDNA repair in atherosclerosis regression. (**A**) Schematic of the chimeric DNMT3A-CH experiment in Figure 6A-F. (**B**) Total plasma cholesterol. (**C**) Body weight. (**D**) Blood WBCs and percentage of lymphocytes, neutrophils, and monocytes. (**E**) Peripheral blood was assayed by flow cytometry for the percentage of GFP^+ and $GFP^–$ WBCs. (**F**) Aortic root sections were quantified for 8-OHdG in non-Mac2+ areas. (**G**) Total number of macrophages per lesion. For B-G, *n* = 7-9 mice/group. Data are shown as mean ± s.e.m., and *P* values were determined by Student's t-test for panels B-D or two-way ANOVA with Fisher's LSD post-hoc analysis for panels E-G. *P < 0.05; ****P < 0.0001; ns, non-significant.

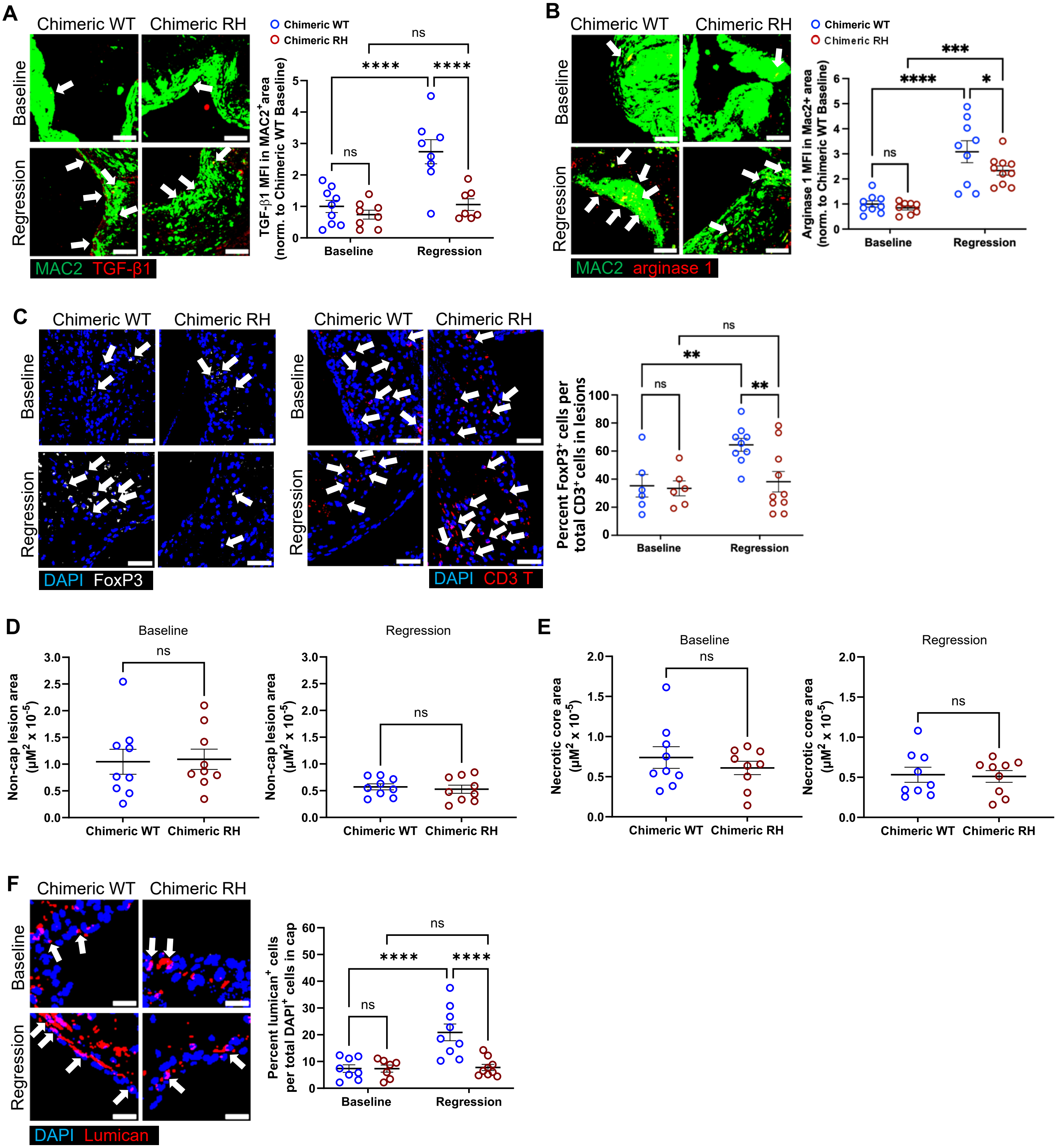

A
Chimeric WT
Chimeric RH
Baseline
Regression
MAC2 TGF-β1
Chimeric WT
Chimeric RH
TGF-β1 MFI in MAC2+area (norm. to Chimeric WT Baseline)
ns
****
****
ns
Baseline
Regression
B
Chimeric WT
Chimeric RH
Baseline
Regression
MAC2 arginase 1
Chimeric WT
Chimeric RH
Arginase 1 MFI in Mac2+ area (norm. to Chimeric WT Baseline)
***
****
*
ns
Baseline
Regression
C
Chimeric WT
Chimeric RH
Baseline
Regression
DAPI FoxP3
Chimeric WT
Chimeric RH
Baseline
Regression
DAPI CD3 T
Percent FoxP3+ cells per total CD3+ cells in lesions
ns
**
ns
**
Baseline
Regression
D
Baseline
Non-cap lesion area (μM2 x 10-5)
ns
Chimeric WT
Chimeric RH
Regression
Non-cap lesion area (μM2 x 10-5)
ns
Chimeric WT
Chimeric RH
E
Baseline
Necrotic core area (μM2 x 10-5)
ns
Chimeric WT
Chimeric RH
Regression
Necrotic core area (μM2 x 10-5)
ns
Chimeric WT
Chimeric RH
F
Chimeric WT
Chimeric RH
Baseline
Regression
DAPI Lumican
Percent lumican+ cells per total DAPI+ cells in cap
ns
****
****
ns
Baseline
Regression

Supplemental Figure 7. Additional supplemental data related to the effect of DNMT3A-CH on oxDNA repair in atherosclerosis regression. (**A**) The sections were immunostained for Mac2 and TGF-β1 (arrows) and quantified as TGF-β1 MFI in $Mac2^+$ areas. Scale bar, 200 μm. *n* = 7-10 mice/group. (**B**) The sections were immunostained for Mac2 and arginase 1 (arrows) and quantified as arginase 1 MFI in $Mac2^+$ areas. Scale bar, 200 μm. *n* = 7-10 mice/group. (**C**) The sections were immunostained for FoxP3 and CD3 (arrows) and stained for DAPI, and the percentage of $FoxP3^+$ of total CD3+ T cells was quantified (n = 7-10 mice/group). Scale bar, 200 μm. (**D-E**) Non-cap lesion area and necrotic core area of the aortic root lesions were quantified. (**F**) The sections were immunostained for lumican (arrows) and stained for DAPI, and the percentage of $lumican^+$ cells in the fibrous cap areas was quantified. Scale bar, 200 μm. *n* = 7-10 mice/group. Data are shown as mean ± s.e.m., and *P* values were determined by Student's t-test for panels D or two-way ANOVA with Fisher's LSD post-hoc analysis for panels A-C and F. *P < 0.05; **P < 0.01; ***P < 0.001; ****P < 0.0001; ns, non-significant.

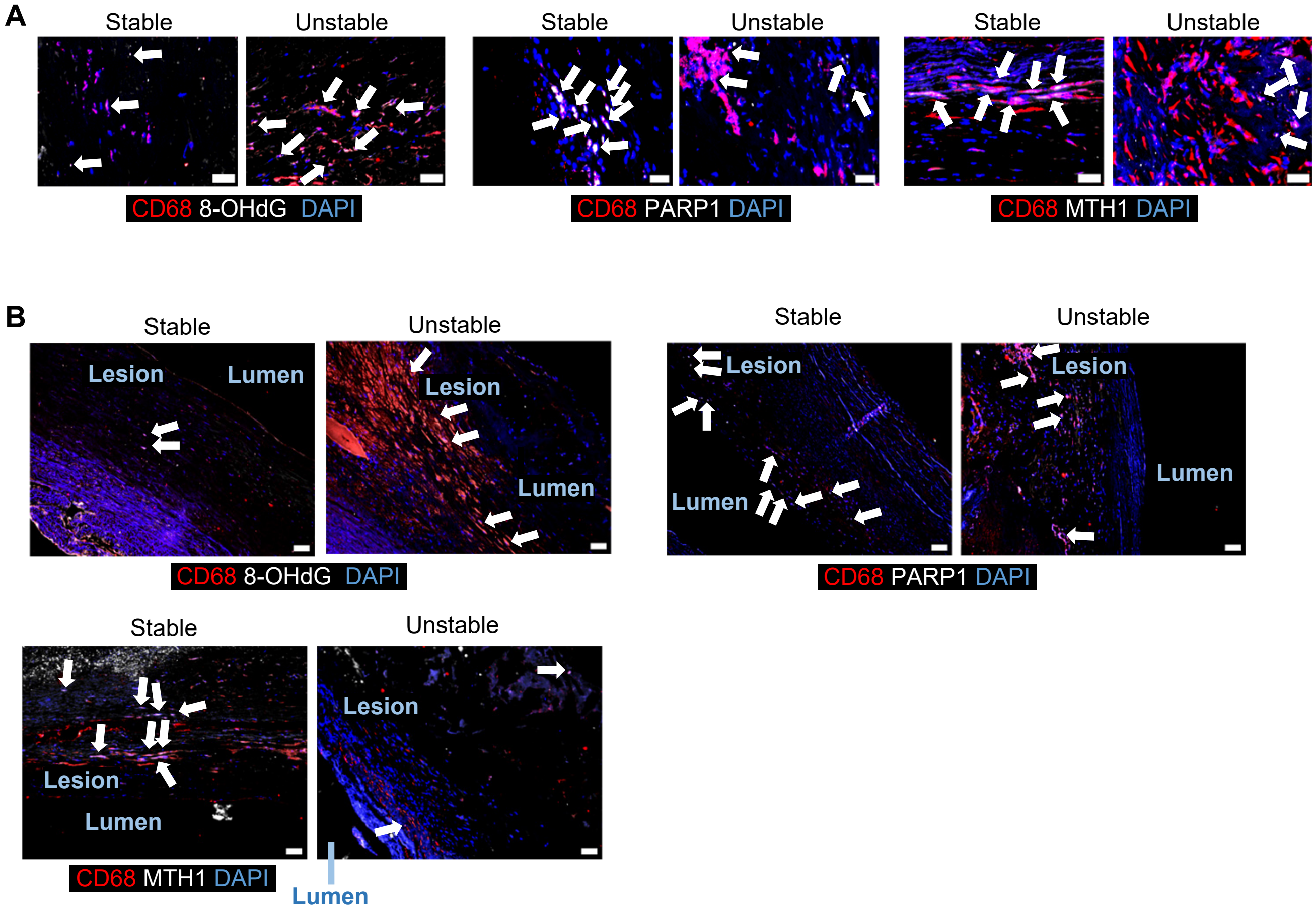

Supplemental Figure 8. Supplemental data related to the oxDNA repair in human carotid atheroma. (**A**) Representative images from Figure 6G. Arrows depict CD68⁺ macrophages with nuclear 8-OHdG, PARP1, or MTH1. Scale bar, 50 µm. (**B**) Lower magnification showing the 8-OHdG, PARP1, and MTH1 expression pattern in the atheroma, with lesion and arterial lumen marked for orientation.

Supplementary Table 1. Primers used for qPCR.

**Gene name**	**Forward Primer (5' to 3')**	**Reverse Primer (5' to 3')**	**Species**
Hprt	TCAGTCAACGGGGGACATAAA	GGGGCTGTACTGCTTAACCAG	mouse
Rplp0	GGACCCGAGAAGACCTCCTT	GCACATCACTCAGAATTTCAATGG	mouse
Nudt1	ACCTCCAGGCTTTATACCCTTG	CTTAGCCCCATCCTCAATGGT	mouse
Parp1	GCTTTATCGAGTGGAGTACGC	GGAGGGAGTCCTTGGGAATAC	mouse
DNMT3A	AGTACGACGACGACGGCTA	CACACTCCACGCAAAAGCAC	human
HPRT1	CCTGGCGTCGTGATTAGTGAT	AGACGTTCAGTCCTGTCCATAA	human

REFERENCE

1. Fredman G, Hellmann J, Proto JD, Kuriakose G, Colas RA, Dorweiler B, et al. An imbalance between specialized pro-resolving lipid mediators and pro-inflammatory leukotrienes promotes instability of atherosclerotic plaques. *Nature Communications*. 2016;7:12859.
