# Supplementary material for: Efferocytosis activates a DNMT3A-mediated oxidized DNA repair pathway to enable tissue resolution
Source: J Clin Invest. 2026 Jul 21;136(20):e203265. doi: 10.1172/JCI203265 (PMC13668614; doi:10.1172/JCI203265)

Fig.2F

PARP1

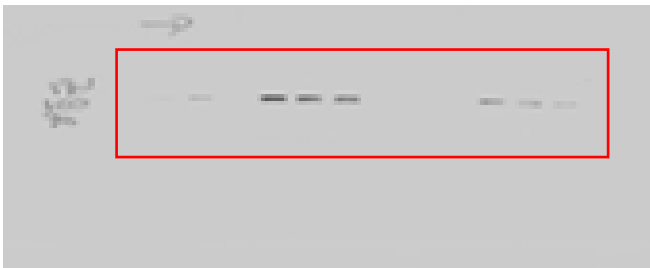

Poly/Mono-ADP ribose or PAR chains

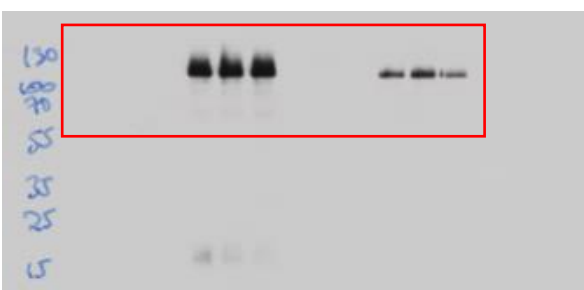

Histone 3

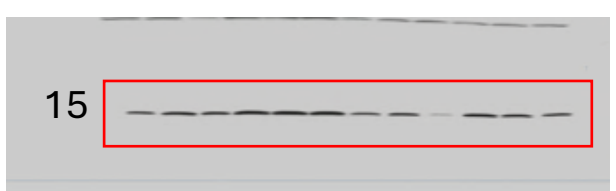

Fig.2G

PARP1

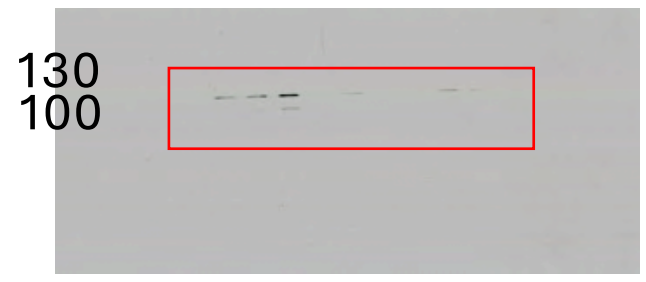

Histone 3

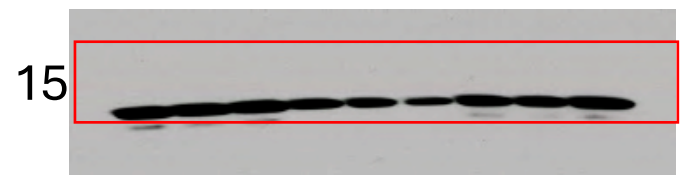

Fig.3B

Lamin B1

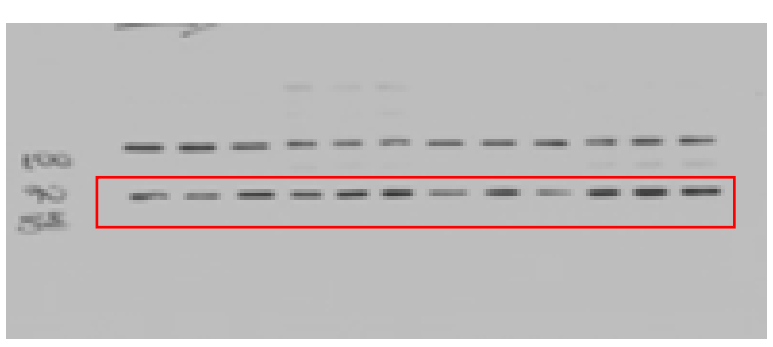

MTH1

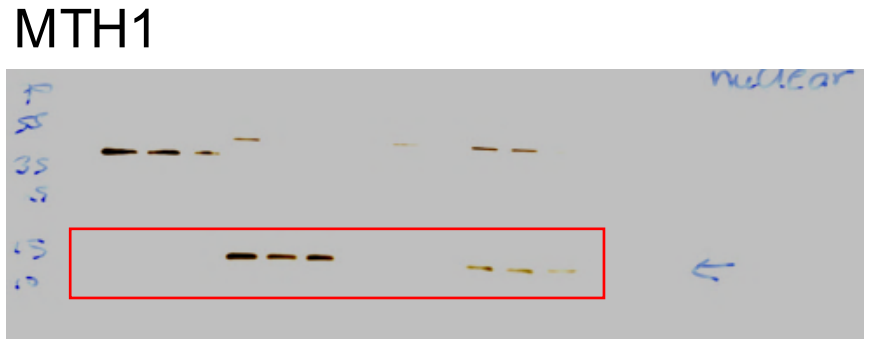

Fig.3C

Lamin B1

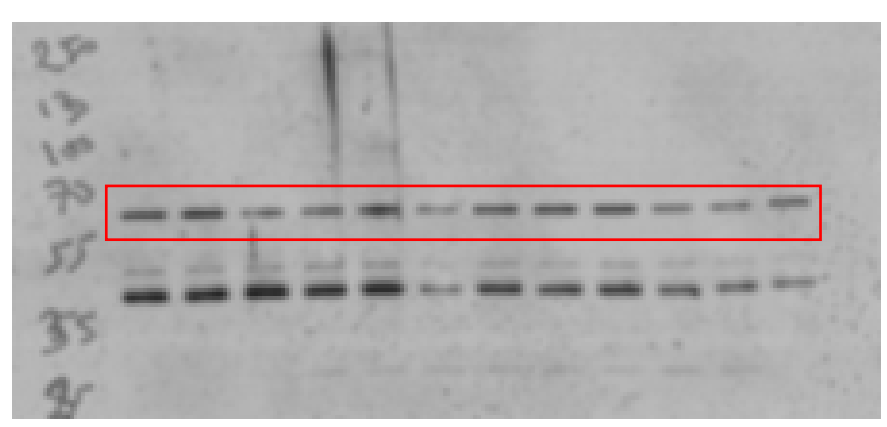

MTH1

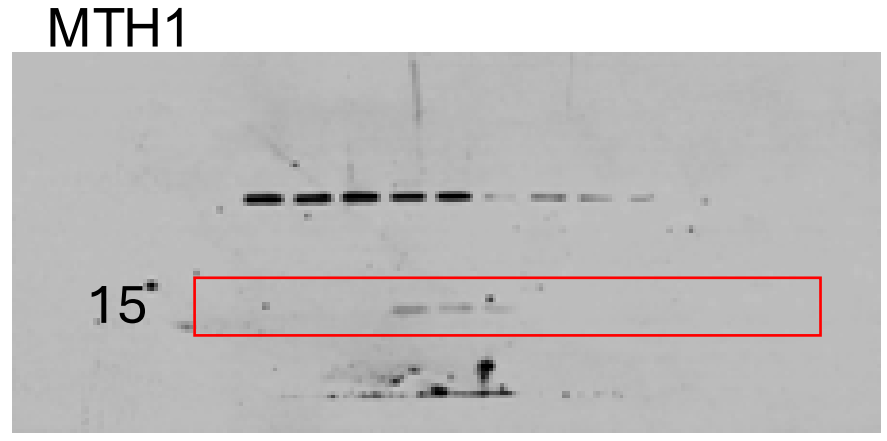

Fig.3F

Lamin B1

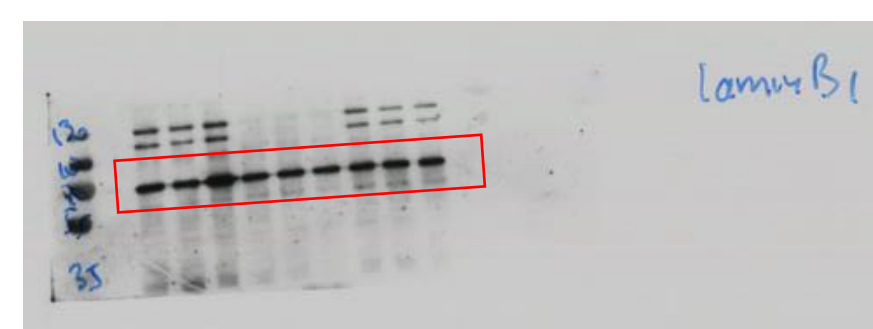

MTH1

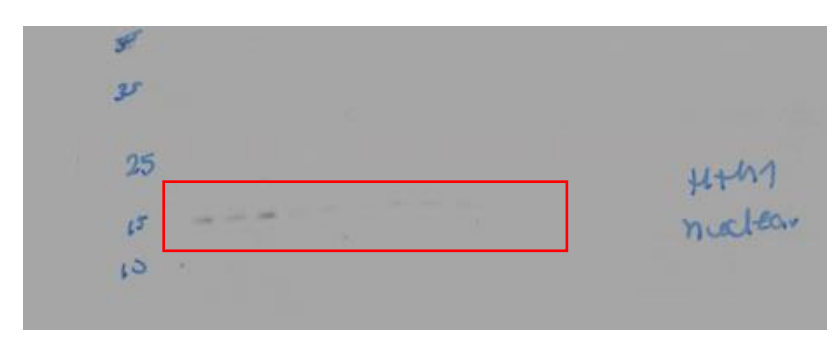

Supplement: Supplementary file 2 [file jci-136-203265-s030.pdf]
